# Recent Discovery of Heterocyclic Alkaloids from Marine-Derived *Aspergillus* Species

**DOI:** 10.3390/md18010054

**Published:** 2020-01-14

**Authors:** Kuo Xu, Xiao-Long Yuan, Chen Li, Xiao-Dong Li

**Affiliations:** 1 Tobacco Research Institute of Chinese Academy of Agricultural Sciences, Qingdao 266101, China; xukuoworld@126.com (K.X.); yuanxiaolong@caas.cn (X.-L.Y.); 2 Yantai Institute of Coastal Zone Research, Chinese Academy of Sciences, Yantai 264003, China; Lychees6601@163.com; 3 Key Laboratory of marine biotechnology in Universities of Shandong (Ludong University), School of Life Sciences, Ludong University, Yantai 264025, China

**Keywords:** *Aspergillus*, metabolite, marine, alkaloid, biological activity

## Abstract

Nitrogen heterocycles have drawn considerable attention due to of their significant biological activities. The marine fungi residing in extreme environments are among the richest sources of these basic nitrogen-containing secondary metabolites. As one of the most well-known universal groups of filamentous fungi, marine-derived *Aspergillus* species produce a large number of structurally unique heterocyclic alkaloids. This review attempts to provide a comprehensive summary of the structural diversity and biological activities of heterocyclic alkaloids that are produced by marine-derived *Aspergillus* species. Herein, a total of 130 such structures that were reported from the beginning of 2014 through the end of 2018 are included, and 75 references are cited in this review, which will benefit future drug development and innovation.

## 1. Introduction

Heterocyclic alkaloids are one of the most challenging natural product classes to characterize, not only because of their structurally unique skeletons that arise from distinct amino acids, but also because of their potential bioactivities. These nitrogen heterocycles are among the most active molecules and they are currently in various phases of human clinical trials for treating various diseases [1,2,3]. The ocean is a rich underexploited source of novel and bioactive molecules, because extreme marine conditions, including low temperature, high pressure, reduced light, and the presence of predators, can cause marine organisms to develop machinery for the construction of a greater diversity of metabolites than terrestrial organisms [4,5,6,7,8]. Fungi living in the extreme environments that are typical of marine ecosystems are very sensitive to culture media and are more liable to produce novel metabolites than fungi living in less extreme environments. As one of the most well-known universal groups of filamentous fungi, marine-derived *Aspergillus* species have one of the main sources of new heterocyclic alkaloids in recent years. This mini-review attempts to provide a comprehensive summary of the structural diversity and biological activities of the nitrogen-containing secondary metabolites that are produced by marine-derived *Aspergillus* species.

A series of excellent reviews on various aspects of secondary metabolites that are derived from the genus *Aspergillus* have been published in the past five years (from 2014 to present) [9,10,11,12,13,14,15,16]. However, there is only one work specifically aimed at *Aspergillus* species from the marine environment. In 2018, K.W. Wang and P. Ding summarized the information on 232 new bioactive secondary metabolites from marine-derived *Aspergillus* species, which had been reported from 2006 to 2016, with classification on the basis of biological activity and chemical structure [12]. As part of our ongoing investigations of biological compounds from endophytic *Aspergillus* species that reside on the marine brown algae *Leathesia nana* (Chordariaceae) [17], a detailed and comprehensive literature survey disclosed that the previously published structures might not be adequately represented. To the best of our knowledge, a total of approximately 400 new compounds were isolated from marine-derived *Aspergillus* species from the beginning of 2014 to the end of 2018 (see Supplementary Materials), of which 130 could be classified as heterocyclic alkaloids. This review aims to provide an update on the recent discoveries of the heterocyclic alkaloids that are produced by marine-derived *Aspergillus* species.

The selection of original articles was of greatest importance because these papers had a direct impact on the findings and the final results. This review included all original articles registered with the relevant subject in the Web of Science Core Collection database between 2014 and 2018. The literature search was performed while using a previously reported search method [18,19]. The search strategy was as follows: “Title: (from *Aspergillus*); Refined by: Topic (marine) and Document types (article); Timespan: 2014–2018; Indexes: SCI-EXPANDED, CPCI-S”. Notably, the present work was preliminarily planned in April 2019, and the studies that were published or being submitted in the current year might not be accurately indexed in the Web of Science Core Collection database; thus, the timespan of the literature search was from 2014 to 2018. With this approach, 166 records were finally identified and were considered to cover most of the related studies.

After retrieving the records that were related to the field of natural product chemistry, 123 original articles were indexed in the Web of Science Core Collection database over a period of five years (from the beginning of 2014 to the end of 2018). During this period, 398 naturally occurring compounds were isolated and characterized from marine-derived *Aspergillus* species. The 130 nitrogen-heterocyclic compounds included accounted for 32.7% of all newly reported secondary metabolites. Supplementary Materials lists all 123 original articles and the structures of these 398 newly reported secondary metabolites. This critical review focuses on the structural diversity, biological activities, and sources of these newly reported heterocyclic alkaloids.

## 2. Structural Diversity

**Figure 1:**
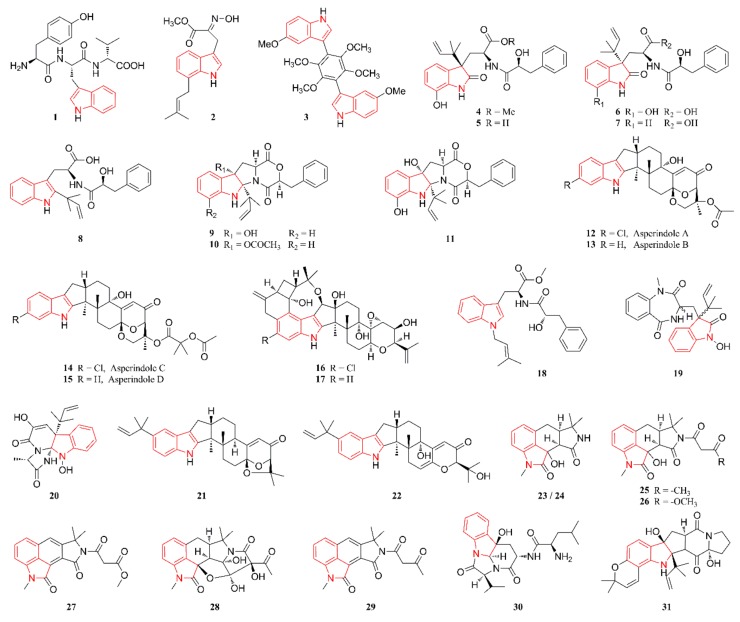
Indole alkaloids produced by marine-derived *Aspergillus* species (**1**–**31**).

**Figure 2:**
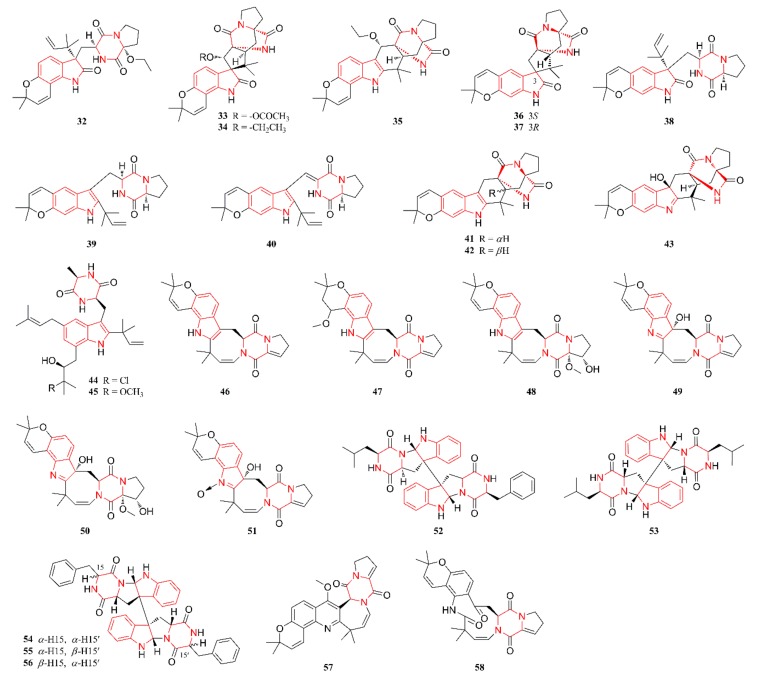
Diketopiperazine alkaloids produced by marine-derived *Aspergillus* species (**32**–**58**).

**Figure 3:**
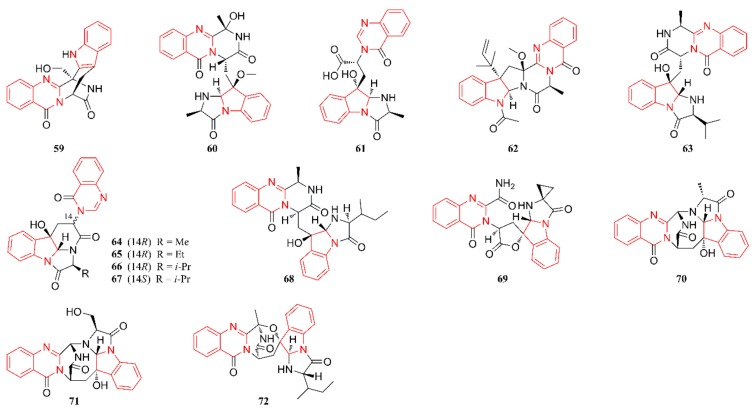
Quinazoline alkaloids produced by marine-derived *Aspergillus* species (**59**–**72**).

**Figure 4:**
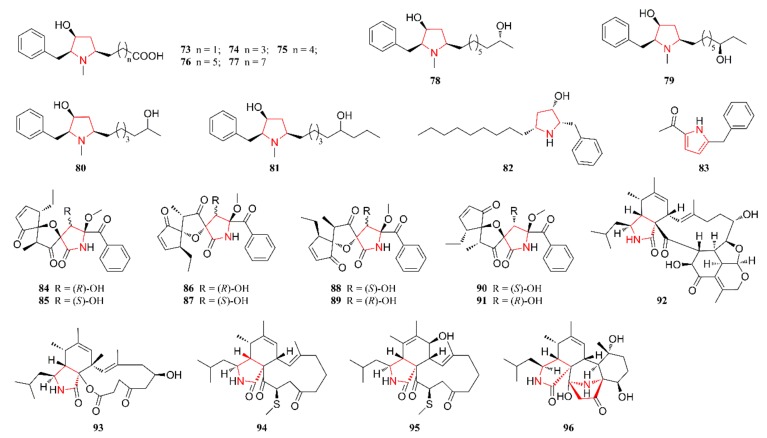
Pyrrolidine alkaloids produced by marine-derived *Aspergillus* species (**73**–**96**).

**Figure 5:**
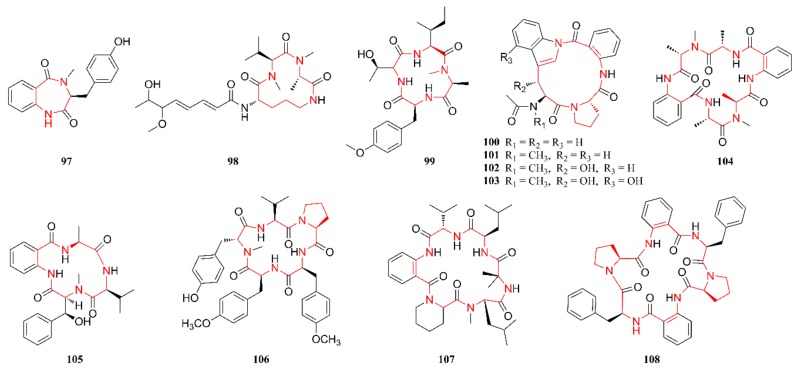
Cyclic peptide alkaloids produced by marine-derived *Aspergillus* species (**97**–**108**).

**Figure 6:**
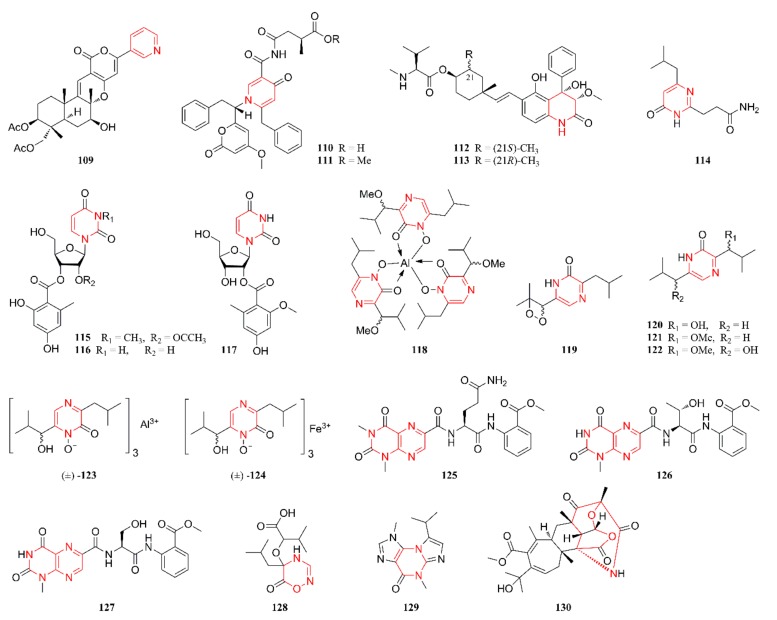
Other alkaloids isolated from marine-derived *Aspergillus* species (**109**–**130**).

Figure 1, Figure 2, Figure 3, Figure 4, Figure 5 and Figure 6 present the structures of newly reported heterocyclic alkaloids (**1**–**130**) produced by marine-derived *Aspergillus* species from 2014 to 2018, in which the nitrogen-containing heterocyclic rings are marked in red. These heterocyclic alkaloids could be classified into six major categories: indole alkaloids (**1**–**31**), diketopiperazine alkaloids (**32**–**58**), quinazoline alkaloids (**59**–**72**), pyrrolidine alkaloids (**73**–**96**), cyclopeptide alkaloids (**97**–**108**), and other heterocyclic alkaloids (**109**–**130**) based on their structural patterns.

### 2.1. Indole Alkaloids

Indole alkaloids serve as the active moiety in several clinical drugs, such as reserpine, and several well-known drugs, such as sumatriptan, tadalafil, fluvastatin, and rizatriptan, were designed on the basis of the indole framework [20]. The indole moiety is present in a wide range of marine natural products, especially fungal metabolites [8,21,22]. Figure 1 lists the structures of indole alkaloids produced by marine-derived *Aspergillus* species. Compound **1** was isolated from a culture broth of a gorgonian-originating fungal strain, *A.* sp. SCSIO 41501, and then characterized as a new linear peptide with three amino acid residues, d-Tyr, d-Val, and l-Trp [23]. Compound **2** was obtained from the coral-associated fungus *A. terreus*, whose structure featured an unusual (*E*)-oxime group, which is rare in natural products [24]. The structure of compound **3** was defined and characterized as a previously unreported bis-indolyl benzenoid and it was isolated from cultures of the marine sponge-derived fungus *A. candidus* KUFA0062 [25]. The C-3 position of the indole fragment in compounds **1**–**3** was substituted by methylene and phenyl groups, which were considered the same type of substituent. Chemical investigation of the algal-derived endophytic fungus *A. alabamensis* EN-547 led to the isolation of two new compounds, **4** and **5**, possessing a rare diketomorpholine fragment [26]. The rice-based culture of a marine-associated fungal strain *A.* sp. MEXU 27854 was extensively chromatographed to produce five dioxomorpholine derivatives **6**–**10** [27]. The structure of compound **11**, which was obtained from a marine sediment-derived *Aspergillus* sp. CMB-M081F, was identified and characterized as a dioxomorpholine derivative [28]. Compounds **12**–**15** were isolated from the fungal strain *Aspergillus* sp. from an unidentified colonial ascidian and then characterized as four new indole-diterpene alkaloids [29]. Compounds **16** and **17** were isolated and identified from the cultures of the endophytic fungus *A. nidulans* EN-330 that were collected from the marine red alga *Polysiphonia scopulorum* [30]. Interestingly, the structures of compounds **12** and **14**–**16** contain a halogen atom, which is rare in fungal secondary metabolites. Compound **18** represents the first example of an *N*-isopentenyl tryptophan methyl ester with a phenyl propanoic amide arm and it was identified from the marine sponge-derived fungus *A.* sp. SCSIO XWS03F03 [31]. Compound **19** was obtained through chromatographic separation of the crude organic extract of a sponge-associated fungal strain *A.* sp., whose structure was a tryptophan-derived indole alkaloid [32]. Compound **20** was characterized as a new polycyclic alkaloid, which was isolated by further chemical investigation of a coral-associated fungal strain *A. versicolor* LZD-14-1 [33]. Compounds **21** and **22** were identified and characterized as two new indole diterpenoids and they were isolated from the fermentation broth of the fungal strain *A. flavus* OUCMDZ-2205 [34]. Compounds **23**–**30** were reported as eight new cyclopiazonic acid (CPA)-type alkaloids, which are usually composed of three structural units: an indole, a tetramic acid unit, and a malonic acid unit. More precisely, compounds **23**–**29** were isolated from the culture of an epiphytic fungal strain of *A. oryzae* residing in marine sediments that were collected from Langqi Island, China, while compound **30** was obtained from the culture of a marine isopod-associated endophytic fungus, *A.* sp. Z-4, which was derived from the marine isopod *Ligia oceanica* [35,36,37]. Compound **31** was characterized as a new prenylated indole alkaloid, which was isolated from a coculture of the marine-derived fungi *A. sulphureus* KMM 4640 and *Isaria felina* KMM 4639 [38].

### 2.2. Diketopiperazine Alkaloids

Diketopiperazine alkaloids are common metabolites of microorganisms that are widely distributed in filamentous fungi, especially in the genera *Aspergillus* and *Penicillium* of the phylum Ascomycota or sac fungi [39]. Interestingly, an indole fragment is typically present in the structures of these diketopiperazine alkaloids. Figure 2 lists the structures of diketopiperazine alkaloids that are produced by marine-derived *Aspergillus* species. Compounds **32**–**35** were isolated from a coculture of the marine sediment-derived fungi *A. sulphureus* KMM 4640 and *Isaria felina* KMM 4639 and identified as four new prenylated indole diketopiperazine alkaloids [38]. Compounds **36**–**43** were characterized as eight linearly fused prenylated indole diketopiperazines featuring an unusual pyrano[3,2-f]indole unit, which were isolated from the culture of a fungal strain, *A. versicolor*, residing in mud from the South China Sea [40]. Compounds **44** and **45** were isolated from the Antarctic marine-derived *A.* sp. SF-5976 obtained from an unidentified marine organism that was collected in the Ross Sea [41]. Compounds **46**–**51** were identified as six new prenylated indole diketopiperazines and they were isolated from a culture of the marine sediment-derived fungus *A. versicolor* HDN08-60 [42]. These compounds are characterized by a 6/6/5/8/6/5 hexacyclic ring system that possesses a hydrogenated azocine unit. Compounds **52**–**56** were reported as four new bis-indole diketopiperazine alkaloids characterized by the presence of two indole diketopiperazines in their structures. More specifically, compounds **52** and **53** were isolated from an organic extract of the sponge-derived fungal strains *A.* sp. SF-5280 and *A. violaceofuscus*, respectively, while compounds **54**–**56** were from a culture of a fungus residing in marine shrimp that were collected along the coast of Dinghai, China [43,44,45]. Compounds **57** and **58** were also isolated from a culture of the marine sediment-derived fungus *A. versicolor* HDN08-60, and their structures featured the presence of only a diketopiperazine fragment, but no indole ring. Structurally, compound **57** possesses an unprecedented skeleton of a 2,5-dihydro-1*H*-azepino[4,3-b]quinoline system, while **58** contains a novel 6/6/11/6/5 pentacyclic ring system. Moreover, the former compound is considered to be a derivative of the latter [42].

### 2.3. Quinazoline Alkaloids

Figure 3 lists the structures of quinazoline alkaloids that are produced by marine-derived *Aspergillus* species. A quinazoline moiety was found in all these alkaloids, which might provide insights into the biogenetic relationships of quinazoline-containing indole alkaloids. Compounds **59**–**61** were characterized as two new quinazoline alkaloids and they were isolated from a culture of the deep-sea-derived fungus *A. fumigatus* SCSIO 41012 [46]. Compound **62** originated from a culture of the Australian marine sediment-derived *A.* sp. CMB-M081F, whose structure was elucidated by detailed spectroscopic analysis and biosynthetic considerations [28]. A solid-substrate culture of strain *A.* sp. F452 residing in submerged decaying wood was extensively chromatographed to produce five new quinazoline-containing alkaloids **63**–**67**. Among them, compound **63** represents a new member of the fumiquinazoline class of alkaloids, which has been reported in a number of marine-derived *Aspergillus*, *Acremonium*, and *Scopulariopsis* fungal strains [47]. Compounds **68**–**72** were characterized as six new polycyclic alkaloids and they were isolated from a culture of a coral-associated fungus, *A. versicolor* LZD-14-1 [33].

### 2.4. Pyrrolidine Alkaloids

Figure 4 lists the structures of pyrrolidine alkaloids that are produced by marine-derived *Aspergillus* species. Bioactivity-guided chemical investigation of cultures of the marine sponge-associated fungal strain *A. flocculosus* 16D-1 facilitated the isolation of nine pyrrolidine alkaloids **73**–**81**. The structures and configurations of these compounds were elucidated by detailed spectroscopic analysis, the modified Mosher’s method, and comparisons with literature data [48]. Compound **82** was isolated and characterized as a new hydroxypyrrolidine alkaloid from cultures of the marine sponge-associated fungus *A. candidus* KUFA 0062 [25]. Compound **83** was produced by a coculture of gorgonian-derived fungal strains of *A. sclerotiorum* and *P. citrinum* and characterized as a pyrrole analog [49]. Compounds **84**–**91** were identified and characterized as having a spiro-heterocyclic γ-lactam skeleton and they were isolated from a culture broth of the marine fish-associated endophytic fungi *A. fumigatus* [50]. Compounds **92**–**96** were characterized as four aspochalasin analogs and they were obtained from the intestines of the marine isopod *Ligia oceanica*, collected along the coast of Dinghai in Zhoushan, Zhejiang Province, China [51,52,53]. Aspochalasins are a small group of cytochalasans structurally featuring a macrocyclic ring system and perhydroisoindol-1-one unit with an isobutyl side chain. Among them, the structure of compound **92** includes a unique 5/6/6 tricyclic ring fused with the skeleton of aspochalasin [51]. Compounds **94** and **95** represent the first thiomethyl-substituted aspochalasin analogs [52]. Compound **96** is rare, in that it contains two nitrogen atoms in its molecular structure and an unusual skeleton that includes an azabicyclo moiety [53].

### 2.5. Cyclopeptide Alkaloids

Cyclopeptide alkaloids are mainly constructed from proteinogenic or nonproteinogenic amino acids that are joined together by amide bonds [54]. These alkaloids can be widely synthesized by both terrestrial and marine organisms. A diversity of cyclopeptide alkaloids with intriguing structures and possible pharmaceutical activities has been identified from marine fungi, a well-known producer [55]. To the best of our knowledge, twelve cyclic peptides (**97**–**105**) were published from 2014 to 2018. Figure 5 lists the structures of cyclopeptide alkaloids that are produced by marine-derived *Aspergillus* species. Compound **97** was isolated and characterized as a novel cyclic dipeptide with a skeleton of cyclo-(anthranilic acid-l-*N*-Me-Tyr) and it could also be considered a benzodiazepine alkaloid of the cyclopenin group [56]. Compounds **98** and **99** were isolated from the EtOAc extract of an endophytic fungal strain, *A. violaceofuscus*, residing in the interior of the marine sponge *Reniochalina* sp. collected from the Xisha Islands in the South China Sea. The structure of compound **98** was established as an aspochracin-type cyclic tripeptide, while that of **99** was elucidated as a cyclic tetrapeptide with the sequence cyclo-[l-Thr-l-O-Me-Tyr-l-*N*-Me-Ala-l-Ile] [44]. Compounds **100**–**104** were isolated from the fungal strain *A. versicolor* ZLN-60, which was obtained from marine sediment in the Yellow Sea in China. More precisely, the structures of compounds **100**–**103** were established as four cyclic peptides that possess a rare amide linkage between the carboxylic acid in anthranilic acid and the nitrogen in an indole moiety, while that of **104** was an anthranilic acid-containing hexapeptide [57,58]. Compound **105** was elucidated as a new cyclic tetrapeptide with a skeleton of cyclo[anthranilic acid-3(*S*)-OH-*N*-Me-Phe-d-Val-l-Ala] [59]. Compound **106** was isolated from a culture broth of the gorgonian-derived fungus *A. terreus* SCSGAF0162 and was characterized as a cyclic tetrapeptide with a skeleton of cyclo[l-Val-(*N*-Me-)-d-Tyr-(O-Me-)-l-Tyr-(O-Me-)-l-Tyr-l-Pro] [23]. Compound **107** was established as a new cyclohexapeptide with the sequence [cyclo (anthranilic acid-l-Val-d-Leu-l-Ala-*N*-methyl-l-Leu-d-pipecolic acid)] and it was isolated from the sponge-derived fungus *A. similanensis* KUFA 0013. Its amino acid sequence was the same as that of a previously reported compound (PF1171C), but the absolute configuration was different [60]. Compound **108** was isolated from the gorgonian-associated fungus *A. versicolor* TA01-14 that was collected from the South China Sea. Its structure was identified and characterized as a centrally symmetric cyclohexapeptide with a skeleton of cyclo [l-Phe-(anthranilic acid)-l-Pro-l-Phe-(anthranilic acid)-l-Pro] [61].

### 2.6. Other Heterocyclic Alkaloids

The remaining heterocyclic alkaloids (**109**–**130**) that were produced by marine-derived *Aspergillus* species are summarized in this section, and their structures are listed in Figure 6. Compounds **109**–**111** were identified and characterized as three new pyridine derivatives. In contrast, compound **109** was obtained from the EtOAc extract of cultures of the marine sponge-derived fungal strain *A. similanensis* KUFA 0013, while compounds **110** and **111** were identified from the cultures of the marine alga-derived fungus *A. niger* SCSIO Jcsw6F30 [60,62]. Compounds **112** and **113** were characterized as two new prenylated dihydroquinolone derivatives that were isolated from the mycelia of a gorgonian-derived *Aspergillus* fungus and they represent the first examples of prenylated dihydroquinolone derivatives containing an amino acid residue in the side chain [63]. Compounds **114** and **115** were isolated from the cultures of the marine sediment-derived epiphytic fungi *A. versicolor* and *A. flavus* KMM 4650, respectively, while compounds **116** and **117** were identified from the gorgonian-derived endophytic fungus *A. versicolor* [64,65,66]. Structurally, compounds **114**–**117** represent four pyrimidine derivatives, and compounds **115**–**117** are aromatic nucleosides. Compounds **118**–**122** were isolated from the fermentation broth of the marine coral-derived halotolerant *A. ochraceus* LCJ11-102 that was cultivated in nutrient-limited medium containing 10% NaI. Compounds **123** and **124** were isolated from a coculture of the marine gorgonian-derived *P. citrinum* SCSGAF 0052 and *A. sclerotiorum* [49,67]. These seven heterocyclic alkaloids were characterized as new pyrazinone alkaloids. The structures of compounds **125**–**127** were characterized as three open-chain peptides with an unusual skeleton of 1,3-dimethyllumazine-6-carboxylic acid, coupled to glutamine and anthranilic acid methyl ester [68,69]. In contrast, compound **125** was isolated from a culture of the mangrove-derived fungal strain *A.* sp. (33241) [68], while compounds **126** and **127** were obtained from cultures of the marine sediment-derived fungal strain *A. terreus* FA009 [69]. Compound **128** was identified as a novel oxadiazin derivative and it was also isolated from a coculture of the marine gorgonian-derived *P. citrinum* SCSGAF 0052 and *A. sclerotiorum* [49]. Compound **129** was isolated and identified from a static culture of the marine sediment-originated fungus *A. sydowii* SP-1 and possesses a 1*H*-imidazo[2,1-b]purin-4(5*H*)-one skeleton [70]. Compound **130** was elucidated as a novel hybrid polyketide-terpenoid with a unique skeleton of fused polycyclic fragments and it was isolated and identified from a crab collected from a Kueishantao hydrothermal vent in China [71].

## 3. Production Environment

Endophytic and epiphytic fungi have proven to be prolific sources of bioactive natural products with unique structures and potent pharmaceutical activity; such fungi harmoniously colonize the internal tissues of their hosts usually without causing obvious damage to the hosts [72,73]. Marine-derived fungi could be isolated from every possible marine habitat, such as marine sediments, marine invertebrates (sponges, corals, ascidians, and holothurians), and vertebrates (mainly fish), as well as marine plants (algae, driftwood, and mangrove plants) [74,75]. As shown in Table 1, a total of 44 heterocyclic alkaloids (**11**, **23**–**29**, **31**–**43**, **46**–**51**, **57**–**62**, **97**, **100**–**104**, **114**, **115**, **126**, **127**, and **129**) originated from the fungi residing in marine sediments, which accounted for 33.8% of the 130 nitrogen-heterocyclic secondary metabolites. More precisely, the producing strains of **59**–**61**, **97**, and **114** originated from deep-sea sediment (deeper than 100 m) [46,56,64], while the other strains were collected from marine sediments at depths above 100 m or even tideland mud [28,35,36,40,42,57,65,69,70]. Unfortunately, the source of the producing strain of **31**–**35** (*A. sulphureus* KMM 4640 and *I. felina* KMM 4639) was not described [39]. To the best of our knowledge, an overwhelming majority of these fungal strains were isolated from marine invertebrates, including corals, sponges, crabs, shrimps, ascidians, and some isopods. For instance, a total of 24 heterocyclic alkaloids (**1**, **2**, **20**, **68**–**72**, **83**, **105**, **106**, **108**, **112**, **113**, **116**–**124**, and **128**) were identified from the endophytic fungal strains of marine gorgonian species, including *Melitodes squamata* [23], *Sarcophyton subviride* [24], *Pseudopterogorgia* sp. LZD-14 [33], *Muricella flexuosa* [49], *Echinogorgia aurantiaca* [59], *Carijoa* sp. GX-WZ-2010001 [61], *Muricella abnormaliz* [63], and *Dichotella gemmacea* [66,67], and they accounted for 18.5% of the newly reported heterocyclic alkaloids. A total of 19 heterocyclic alkaloids (**3**, **18**, **19**, **52**, **53**, **73**–**82**, **98**, **99**, **107**, and **109**) were identified from the fungi residing in marine sponges, including *Epipolasis* sp. [25], *Tethya aurantium* [32], *Reniochalina* sp. [44], *Phakellia fusca* [48], *Rhabdermia* sp. [60], and an unidentified species [31,43], and they accounted for 14.6% of the newly reported heterocyclic alkaloids. Six heterocyclic alkaloids (**30** and **92**–**96**) were also isolated from the endophytic fungi (*A.* sp. Z-4) residing in the marine isopod *Ligia oceanica* [37,51,52,53], while only one heterocyclic alkaloid (**130**) was identified from the endophytic fungi obtained from the marine crab *Xenograpsus testudinatus* [71]. Moreover, the producing strains of **21**, **22**, and **54**–**56** originated from marine shrimp or prawns [34,45], while those of compounds **12**–**15** were derived from an unidentified colonial ascidian that was collected at Shikotan Island in the Pacific Ocean [29]. In addition to these producing strains of marine invertebrate origin, only one fungal strain, which produced **84**–**91**, was obtained from the marine fish *Mugil cephalus*, representing vertebrates [50]. It appears that the number of the fungal strains of marine plant origin that produce heterocyclic alkaloids is much less than that of marine invertebrate and vertebrate origin. For example, the producing strains of the heterocyclic alkaloids **4**, **5**, **16**, **17**, **110**, and **111** originated from the fungi residing in marine algal species, including *Ceramium japonicum* [26], *Polysiphonia scopulorum* [30], and *Sargassum* sp. [58,62], while those of **63**–**67** were derived from submerged decaying wood at Jeju Island, Korea [47]. Moreover, the producing strain of 125 originated from the mangrove *Bruguiera sexangula* var. *rhynchopetala* that was collected in the South China Sea [68]. Interestingly, two heterocyclic alkaloids (**44** and **45**) were isolated from the fungi residing in an unidentified marine organism that was collected in the Ross Sea without any detailed description for species identification [41].

## 4. Biological Activities

The biological activities of these heterocyclic alkaloids are detailed in Table 1. Anticancer and antimicrobial activities, as well as anti-inflammatory activity, were the three main indexes that were used to assess the pharmacological activity of these natural heterocyclic alkaloids. In this section, alkaloids with potent biological activities are the focus, and detailed descriptions are provided below.

### 4.1. Anticancer Activities

**Figure 7:**
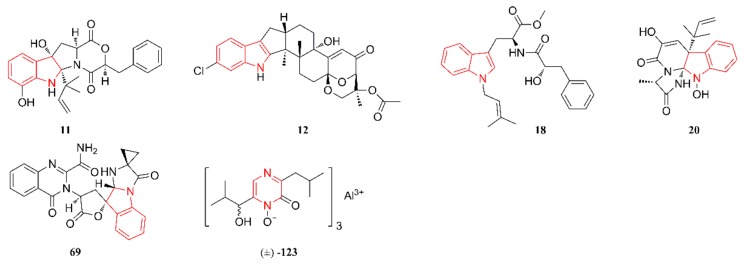
Anticancer heterocyclic alkaloids produced by marine-derived *Aspergillus* species.

Figure 7 lists the anticancer heterocyclic alkaloids. Compound **11** at 20 μM was demonstrated to be a noncytotoxic inhibitor of P-glycoprotein-mediated drug efflux in multidrug-resistant (MDR) human colon cancer cells and it might be used to improve the prognosis for MDR cancer chemotherapy [28]. Compound **12** showed cytotoxicity against human PC-3, LNCaP, and 22Rv1 cells, with IC_50_ values of 69.4 µM, 47.8 µM, and 4.86 µM, respectively. The reference substance (Docetaxel) displayed IC_50_ values of 15.4 nM, 3.8 nM, and 12.7 nM, respectively. This compound was able to induce the apoptosis of 22Rv1 cells at low micromolar concentrations. Cell cycle progression analyses of 22Rv1 cells that were treated with **12** also revealed discrete G2/M-phase arrest [29]. Compound **18** exhibited potent cytotoxic activity against HL-60 and LNCap cells with IC_50_ values of 3.1 and 4.9 µM, respectively, but with no significant cytotoxicity against the rest of the tested cell lines (HepG2, Hela, A375, A549, HT29, SK-BR-3, and MCF-7) [31]. Compounds **20** and **69** exhibited significant inhibitory activities against thioredoxin reductase with IC_50_ values of 12.2 ± 0.7 and 13.6 ± 0.6 µM (the IC_50_ of the positive control curcumin was 25 µM), but weak toxicity against A549 cells (IC_50_ > 10 µM), which suggested that these two compounds might act as microenvironmental regulators of tumor progression and metastasis [33]. Compound **123** possesses selective cytotoxicity against U937 cells with an IC_50_ value of 4.2 μM and mild cytotoxicity against HeLa and MCF-7 cells, with IC_50_ values of 29.3 μM and 24.8 μM, respectively. The IC_50_ values of doxorubicin (positive control) towards U937, HeLa, and MCF-7 cells were 0.06 μM, 0.8 μM, and 23.1 μM, respectively [49].

### 4.2. Antimicrobial Activities

**Figure 8:**
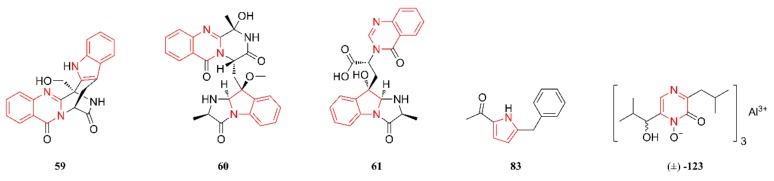
Antimicrobial heterocyclic alkaloids that are produced by marine-derived *Aspergillus* species.

Figure 8 lists the antimicrobial heterocyclic alkaloids. Compounds **59** and **61** were tested for their antimicrobial activities. Compound **59** showed comparable or even higher antibacterial activity than the other tested compounds. This compound also showed excellent antifungal activity against *F. oxysporum* with an MIC of 1.5 µg/mL. Compound **60** exhibited high activity against *S. aureus* (16339 and 29213), with MIC values of 1.565 µg/mL and 0.78 µg/mL, respectively, while compound **61** exhibited significant activity against *A. baumanii* ATCC 19606 with an MIC of 6.25 µg/mL [46]. Although compounds **83** and **123** showed non-significant antimicrobial activity against two common bacterial strains (*S. aureus* and *P. aeruginosa*) and three marine-derived bacteria (*P. nigrifaciens*, *B. amyloliquefaciens*, and *B. stearothermophilus*), they increased the growth of *S. aureus* one-fold at 100 μg/mL, and **123** increased the biofilm formation of *S. aureus* 1.3-fold at 25 μg/mL [49].

### 4.3. Anti-Inflammatory Activities

**Figure 9:**
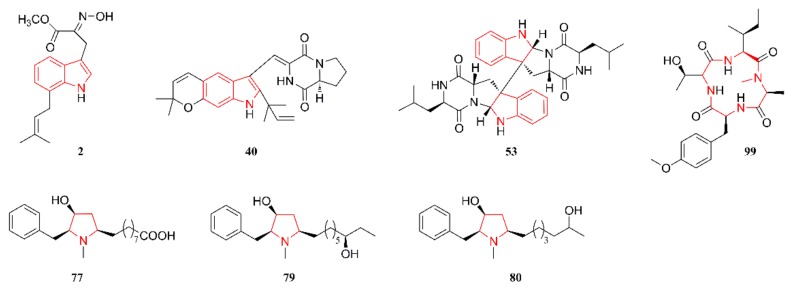
Anti-inflammatory heterocyclic alkaloids produced by marine-derived *Aspergillus* species.

Figure 9 lists the anti-inflammatory heterocyclic alkaloids. Compound **2** showed potent anti-inflammatory activity against NO production with an IC_50_ of 24.64 μM [24]. Compound **40** exhibited an excellent inhibition of iNOS with an IC_50_ of 5.39 μM, but weak activity against Raw 264.7 cells. The inhibitory effects might be the result of cell viability independent of concentration. Molecular docking studies with **40** and iNOS showed that it could adopt an extended conformation and fit well into the ligand binding site of mutant iNOS [40]. Compounds **53** and **99** were evaluated for their inhibitory activities against the production of cytokines in the serum of THP-1 by using the human inflammation cytometric bead array assay. The THP-1 cells that were pretreated with **53** and **99** showed a significant decrease in the LPS-induced expression of IL-10, with inhibitory rates of 78.1% and 84.3% (*p* < 0.01), respectively. Moreover, these two compounds did not show cytotoxicity against THP-1 cells after 24 h of treatment [44]. Compounds **77**, **79**, and **80** showed potent inhibitory activity against IL-6 production, with IC_50_ values of 0.11 μM, 0.19 μM, and 2.3 μM, respectively [48].

### 4.4. Other Biological Activities

**Figure 10:**
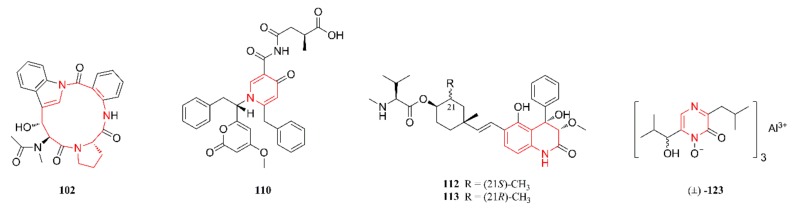
Other bioactive heterocyclic alkaloids produced by marine-derived *Aspergillus* species.

Figure 10 lists other bioactive alkaloids. Compound **102** was found to possess potent lipid-lowering effects, but non-significant cytotoxicity [57]. Compound **110** exhibited significant HIV-1 inhibitory activities against SF162 infection in TZM-bl cells, with IC_50_ and CC_50_ values of 4.7 ± 0.4 and 35.0 ± 2.1 μM (selectivity index of 7.5), respectively, which might be beneficial for the development of heterocyclic alkaloids as anti-HIV agents [62]. Compound **112** showed strong toxicity towards brine shrimp with an LC_50_ value of 6.1 μM as compared with the positive control toosendanin (LC_50_ = 1.73 μM). Compound **113** possessed outstanding anti-RSV activity with an IC_50_ value of 42 nM, being approximately 500-fold stronger than that of the positive control ribavirin (IC_50_ = 20 μM), as well as a higher therapeutic ratio (TC_50_/IC_50_ = 520) [63]. Compound **123** also showed strong toxicity, with an LC_50_ value of 6.1 μM as compared with the positive control toosendanin (LC_50_ = 1.73 μM) [49].

## 5. Conclusions

This review summarized the findings, including the biological activities, on a total of 130 nitrogen-containing secondary metabolites that originate from marine-derived *Aspergillus* species reported from the beginning of 2014 through the end of 2018. All of the original literature in the Web of Science database, which we believe covers most of the newly reported naturally occurring heterocyclic alkaloids from specific sources, was searched. However, several works may not have been retrieved by the literature method used in this review. In the process of preparing this review, compound **23** was reported as speradine B and it was shown to possess an identical planar structure to that of speradine G (**24**), which was not explained in the original articles [35,36]. A careful comparison of the one-dimensional NMR data makes us boldly propose that these two compounds are a pair of diastereoisomers. Further, it is quite interesting that psychrophilin E (**100**) was reported as a new compound by two completely independent research teams in the same year [57,58]. Therefore, for natural product chemists, the research results should be published in a timely manner. At the end of this review, compounds with potent bioactivities were comprehensively described, which will be beneficial in future drug development and innovation.

## Figures and Tables

**Table 1:** The producing strain, biological activities of these heterocyclic alkaloids (**1**–**130**).

NO.	Producing Strain	Environmental Source	Biological Activities	Ref.
**1**	*A.* sp. SCSIO 41501	the gorgonian *Melitodes squamata* collected from the South China Sea, Sanya, China	moderate antiviral activity against HSV-1 under non-cytotoxic concentrations against Vero cells	[23]
**2**	*A. terreus*	the coral *Sarcophyton subviride* collected from the coast of Xisha Island in the South China Sea	potent inhibition on LPS-induced NO production; nonsignificant inhibition on α-Glucosidase	[24]
**3**	*A. candidus* KUFA0062	the marine sponge *Epipolasis* sp. collected at Similan Island National Park (15–20 m), Thailand	weak cytotoxic activity against eight cell lines; nonsignificant antibacterial activity	[25]
**4**	*A. alabamensis* EN-547	the fresh inner tissue of marine alga *Ceramium japonicum* collected at Qingdao, China	moderate antimicrobial activities against *E. coli*, *M. luteus*, *Ed. ictaluri* and *V. alginolyticus*	[26]
**5**	*A. alabamensis* EN-547	the fresh inner tissue of marine alga *Ceramium japonicum* collected at Qingdao, China	moderate antimicrobial activities against *E. coli*, *M. luteus*, *Ed. ictaluri* and *V. alginolyticus*	[26]
**6**	*A.* sp. MEXU 27854	sandy soil collected in the intertidal zone located in Caleta Bay, Acapulco, Guerrero, Mexico	nonsignificant cytotoxic activities	[27]
**7**	*A.* sp. MEXU 27854	sandy soil collected in the intertidal zone located in Caleta Bay, Acapulco, Guerrero, Mexico	no biological activity was tested	[27]
**8**	*A.* sp. MEXU 27854	sandy soil collected in the intertidal zone located in Caleta Bay, Acapulco, Guerrero, Mexico	nonsignificant cytotoxic activities	[27]
**9**	*A.* sp. MEXU 27854	sandy soil collected in the intertidal zone located in Caleta Bay, Acapulco, Guerrero, Mexico	no biological activity was tested	[27]
**10**	*A.* sp. MEXU 27854	sandy soil collected in the intertidal zone located in Caleta Bay, Acapulco, Guerrero, Mexico	no biological activity was tested	[27]
**11**	*A.* sp. CMB-M081F	the marine sediment collected at an intertidal depth of 1 m near Shorncliffe, Queensland, Australia	nonsignificant cytotoxic activities; potent inhibition on P-glycoprotein-mediated drug efflux	[28]
**12**	*A.* sp. KMM 4676	an unidentified colonial ascidian (Shikotan Island, Pacific Ocean)	potent cytotoxicity against 22Rv1, while moderate cytotoxicity against PC-3 and LNCaP	[29]
**13**	*A.* sp. KMM 4676	an unidentified colonial ascidian (Shikotan Island, Pacific Ocean)	no biological activity was tested	[29]
**14**	*A.* sp. KMM 4676	an unidentified colonial ascidian (Shikotan Island, Pacific Ocean)	nonsignificant cytotoxic activities against PC-3, LNCaP and 22Rv1 cell lines	[29]
**15**	*A.* sp. KMM 4676	an unidentified colonial ascidian (Shikotan Island, Pacific Ocean)	no biological activity was tested	[29]
**16**	*A. nidulans* EN-330	The marine red alga *P. scopulorum* var. *villum* collected from Yantai coastline of north China	moderate antimicrobial activities against four human- and aqua-pathogens	[30]
**17**	*A. nidulans* EN-330	The marine red alga *P. scopulorum* var. *villum* collected from Yantai coastline of north China	weak antimicrobial activities against four human- and aqua-pathogens	[30]
**18**	*A.* sp. SCSIO XWS03F03	a sponge collected from the sea area Xuwen County, Guangdong, China	potent cytotoxic activity against HL-60 and LNCap cell lines	[31]
**19**	*A.* sp.	the sponge *Tethya aurantium* (Pallas 1766) collected at the entrance of Limski kanal (a depth of 20 m)	moderate selective activity against marine bacteria; nonsignificant cytotoxicity against L5178Y cells	[32]
**20**	*A. versicolor* LZD-14-1	the gorgonian *Pseudopterogorgia* sp. (LZD-14) collected from the South China Sea	weak cytotoxic activity against A549; potent inhibitory activity against TrxR	[33]
**21**	*A. flavus* OUCMDZ-2205	the prawn *Penaeus vannamei* collected in Lianyungang sea area, Jiangsu Province of China	moderate antibacterial and cytotoxic activities, as well as PKC-beta inhibition	[34]
**22**	*A. flavus* OUCMDZ-2205	the prawn *Penaeus vannamei* collected in Lianyungang sea area, Jiangsu Province of China	moderate cytotoxicity; nonsignificant antibacterial activity and PKC-beta inhibition	[34]
**23**	*A. oryzae*	the marine sediments collected from Langqi Island, Fujian, China	weak cytotoxic activities against Hela and MGC803 cell lines	[35]
**24**	*A. oryzae*	the marine sediments collected from Langqi Island, Fujian, China	nonsignificant cytotoxic activities against Hela, HL-60, and K562 cell lines	[36]
**25**	*A. oryzae*	the marine sediments collected from Langqi Island, Fujian, China	nonsignificant cytotoxic activities against Hela and MGC803 cell lines	[35]
**26**	*A. oryzae*	the marine sediments collected from Langqi Island, Fujian, China	nonsignificant cytotoxic activities against Hela and MGC803 cell lines	[35]
**27**	*A. oryzae*	the marine sediments collected from Langqi Island, Fujian, China	weak cytotoxic activities against Hela and MGC803 cell lines	[35]
**28**	*A. oryzae*	the marine sediments collected from Langqi Island, Fujian, China	nonsignificant cytotoxic activities against Hela, HL-60, and K562 cell lines	[36]
**29**	*A. oryzae*	the marine sediments collected from Langqi Island, Fujian, China	nonsignificant cytotoxic activities against Hela, HL-60, and K562 cell lines	[36]
**30**	*A.* sp Z-4	the marine isopod *Ligia oceanica* collected in Zhoushan, Zhejiang, China	nonsignificant cytotoxicity against PC3 and HCT116	[37]
**31**	*A. sulphureus* KMM 4640 and *I. felina* KMM 4639	marine sediments (no detailed description)	nonsignificant cytotoxic activities against MRC-9, HEK 293T, 22Rv1, PC-3, and LNCaP	[38]
**32**	*A. sulphureus* KMM 4640 and *I. felina* KMM 4639	marine sediments (no detailed description)	nonsignificant cytotoxic activities against MRC-9, HEK 293T, 22Rv1, PC-3, and LNCaP	[38]
**33**	*A. sulphureus* KMM 4640 and *I. felina* KMM 4639	marine sediments (no detailed description)	nonsignificant cytotoxic activities against MRC-9, HEK 293T, 22Rv1, PC-3, and LNCaP	[38]
**34**	*A. sulphureus* KMM 4640 and *I. felina* KMM 4639	marine sediments (no detailed description)	nonsignificant cytotoxic activities against MRC-9, HEK 293T, 22Rv1, PC-3, and LNCaP	[38]
**35**	*A. sulphureus* KMM 4640 and *I. felina* KMM 4639	marine sediments (no detailed description)	nonsignificant cytotoxic activities against MRC-9, HEK 293T, 22Rv1, PC-3, and LNCaP	[38]
**36**	*A. versicolor*	the mud of the South China Sea	moderate inhibitory activities against LPS-induced NO production and iNOS enzyme	[40]
**37**	*A. versicolor*	the mud of the South China Sea	moderate inhibitory activities against LPS-induced NO production and iNOS enzyme	[40]
**38**	*A. versicolor*	the mud of the South China Sea	nonsignificant inhibitory activities against LPS-induced NO production and iNOS enzyme	[40]
**39**	*A. versicolor*	the mud of the South China Sea	moderate inhibitory activities against LPS-induced NO production and iNOS enzyme	[40]
**40**	*A. versicolor*	the mud of the South China Sea	potent inhibitory activities against LPS-induced NO production and iNOS enzyme	[40]
**41**	*A. versicolor*	the mud of the South China Sea	nonsignificant inhibitory activities against LPS-induced NO production and iNOS enzyme	[40]
**42**	*A. versicolor*	the mud of the South China Sea	nonsignificant inhibitory activities against LPS-induced NO production and iNOS enzyme	[40]
**43**	*A. versicolor*	the mud of the South China Sea	nonsignificant inhibitory activities against LPS-induced NO production and iNOS enzyme	[40]
**44**	*A.* sp. SF-5976	an unidentified marine organism collected in the Ross Sea	weak inhibitory activities against LPS-induced NO production in RAW 264.7 and BV2 cells	[41]
**45**	*A.* sp. SF-5976	an unidentified marine organism collected in the Ross Sea	moderate inhibitory activities against LPS-induced NO production in RAW 264.7 and BV2 cells	[41]
**46**	*A. versicolor* HDN08-60	the sediments (at a depth of 35 m) collected in the South China Sea, China	nonsignificant cytotoxic activities against HeLa, HCT-116, HL-60 and K562 cell lines	[42]
**47**	*A. versicolor* HDN08-60	the sediments (at a depth of 35 m) collected in the South China Sea, China	nonsignificant cytotoxic activities against HeLa, HCT-116, HL-60 and K562 cell lines	[42]
**48**	*A. versicolor* HDN08-60	the sediments (at a depth of 35 m) collected in the South China Sea, China	nonsignificant cytotoxic activities against HeLa, HCT-116, HL-60 and K562 cell lines	[42]
**49**	*A. versicolor* HDN08-60	the sediments (at a depth of 35 m) collected in the South China Sea, China	nonsignificant cytotoxic activities against HeLa, HCT-116, HL-60 and K562 cell lines	[42]
**50**	*A. versicolor* HDN08-60	the sediments (at a depth of 35 m) collected in the South China Sea, China	nonsignificant cytotoxic activities against HeLa, HCT-116, HL-60 and K562 cell lines	[42]
**51**	*A. versicolor* HDN08-60	the sediments (at a depth of 35 m) collected in the South China Sea, China	nonsignificant cytotoxic activities against HeLa, HCT-116, HL-60 and K562 cell lines	[42]
**52**	*A.* sp. SF-5280	an unidentified sponge collected at Cheju Island, Korea	moderate inhibitory effects against PTP1B activity	[43]
**53**	*A. violaceofuscus*	the inner part of marine sponge *Reniochalina* sp. collected from Xisha Islands in South China Sea	potent anti-inflammatory activity against IL-10 expression of the LPS-induced THP-1 cells	[44]
**54**	*A.* sp. DX4H	marine shrimp collected in seaside of Dinghai in Zhoushan, Zhejiang Province of China	weak cytotoxic activities against PC3 cell line	[45]
**55**	*A.* sp. DX4H	marine shrimp collected in seaside of Dinghai in Zhoushan, Zhejiang Province of China	weak cytotoxic activities against PC3 cell line	[45]
**56**	*A.* sp. DX4H	marine shrimp collected in seaside of Dinghai in Zhoushan, Zhejiang Province of China	weak cytotoxic activities against PC3 cell line	[45]
**57**	*A. versicolor* HDN08-60	the sediments (at a depth of 35 m) collected in the South China Sea, China	moderate cytotoxic activities and selective PTK inhibitory activities	[42]
**58**	*A. versicolor* HDN08-60	the sediments (at a depth of 35 m) collected in the South China Sea, China	nonsignificant cytotoxic activities against HeLa, HL-60, and K562 cell lines	[42]
**59**	*A. fumigatus* SCSIO 41012	the deep-sea sediments (3614 m) collected from the Indian Ocean	potent antifungal and antibacterial activities	[46]
**60**	*A. fumigatus* SCSIO 41012	the deep-sea sediments (3614 m) collected from the Indian Ocean	potent antibacterial activities	[46]
**61**	*A. fumigatus* SCSIO 41012	the deep-sea sediments (3614 m) collected from the Indian Ocean	potent antibacterial activities	[46]
**62**	*A.* sp. CMB-M081F	the marine sediment collected at an intertidal depth of 1 m near Shorncliffe, Queensland, Australia	nonsignificant cytotoxic activities and inhibition on P-glycoprotein-mediated drug efflux	[28]
**63**	*A.* sp. F452	submerged decaying wood off the shore of Jeju Island, Korea	moderate cytotoxicity; nonsignificant antibacterial activity; weak inhibition against Na+/K+-ATPase	[47]
**64**	*A.* sp. F452	submerged decaying wood off the shore of Jeju Island, Korea	moderate cytotoxicity; nonsignificant antibacterial activity; weak inhibition against Na+/K+-ATPase	[47]
**65**	*A.* sp. F452	submerged decaying wood off the shore of Jeju Island, Korea	moderate cytotoxicity; nonsignificant antibacterial activity; weak inhibition against Na+/K+-ATPase	[47]
**66**	*A.* sp. F452	submerged decaying wood off the shore of Jeju Island, Korea	moderate cytotoxicity; nonsignificant antibacterial activity; weak inhibition against Na+/K+-ATPase	[47]
**67**	*A.* sp. F452	submerged decaying wood off the shore of Jeju Island, Korea	moderate cytotoxicity; nonsignificant antibacterial activity; weak inhibition against Na+/K+-ATPase	[47]
**68**	*A. versicolor* LZD-14-1	the gorgonian *Pseudopterogorgia* sp. (LZD-14) collected from the South China Sea	weak cytotoxic activity against A549; nonsignificant inhibitory activity against TrxR	[33]
**69**	*A. versicolor* LZD-14-1	the gorgonian *Pseudopterogorgia* sp. (LZD-14) collected from the South China Sea	weak cytotoxic activity against A549; potent inhibitory activity against TrxR	[33]
**70**	*A. versicolor* LZD-14-1	the gorgonian *Pseudopterogorgia* sp. (LZD-14) collected from the South China Sea	weak cytotoxic activity against A549; nonsignificant inhibitory activity against TrxR	[33]
**71**	*A. versicolor* LZD-14-1	the gorgonian *Pseudopterogorgia* sp. (LZD-14) collected from the South China Sea	weak cytotoxic activity against A549; nonsignificant inhibitory activity against TrxR	[33]
**72**	*A. versicolor* LZD-14-1	the gorgonian *Pseudopterogorgia* sp. (LZD-14) collected from the South China Sea	weak cytotoxic activity against A549; nonsignificant inhibitory activity against TrxR	[33]
**73**	*A. flocculosus* 16D-1	the inner tissue of marine sponge *Phakellia fusca* collected from Yongxing Island, China	moderate inhibitory activity against IL-6 production in LPS-induced THP-1 cells	[48]
**74**	*A. flocculosus* 16D-1	the inner tissue of marine sponge *Phakellia fusca* collected from Yongxing Island, China	moderate inhibitory activity against IL-6 production in LPS-induced THP-1 cells	[48]
**75**	*A. flocculosus* 16D-1	the inner tissue of marine sponge *Phakellia fusca* collected from Yongxing Island, China	moderate inhibitory activity against IL-6 production in LPS-induced THP-1 cells	[48]
**76**	*A. flocculosus* 16D-1	the inner tissue of marine sponge *Phakellia fusca* collected from Yongxing Island, China	moderate inhibitory activity against IL-6 production in LPS-induced THP-1 cells	[48]
**77**	*A. flocculosus* 16D-1	the inner tissue of marine sponge *Phakellia fusca* collected from Yongxing Island, China	potent inhibitory activity against IL-6 production in LPS-induced THP-1 cells	[48]
**78**	*A. flocculosus* 16D-1	the inner tissue of marine sponge *Phakellia fusca* collected from Yongxing Island, China	moderate inhibitory activity against IL-6 production in LPS-induced THP-1 cells	[48]
**79**	*A. flocculosus* 16D-1	the inner tissue of marine sponge *Phakellia fusca* collected from Yongxing Island, China	potent inhibitory activity against IL-6 production in LPS-induced THP-1 cells	[48]
**80**	*A. flocculosus* 16D-1	the inner tissue of marine sponge *Phakellia fusca* collected from Yongxing Island, China	potent inhibitory activity against IL-6 production in LPS-induced THP-1 cells	[48]
**81**	*A. flocculosus* 16D-1	the inner tissue of marine sponge *Phakellia fusca* collected from Yongxing Island, China	moderate inhibitory activity against IL-6 production in LPS-induced THP-1 cells	[48]
**82**	*A. candidus* KUFA0062	the marine sponge *Epipolasis* sp. collected at Similan Island National Park (15–20 m), Thailand	weak cytotoxic activity against eight cell lines; nonsignificant antibacterial activity	[25]
**83**	*A. sclerotiorum* and *P. citrinum*	the gorgonian *Muricella flexuosa* collected from the South China Sea, Sanya, Hainan Province, China	moderate brine shrimp lethality; nonsignificant antibacterial and anti-biofilm activities	[49]
**84**	*A. fumigatus*	the marine fish *Mugil cephalus*	cytotoxic tests are in progress	[50]
**85**	*A. fumigatus*	the marine fish *Mugil cephalus*	cytotoxic tests are in progress	[50]
**86**	*A. fumigatus*	the marine fish *Mugil cephalus*	cytotoxic tests are in progress	[50]
**87**	*A. fumigatus*	the marine fish *Mugil cephalus*	cytotoxic tests are in progress	[50]
**88**	*A. fumigatus*	the marine fish *Mugil cephalus*	cytotoxic tests are in progress	[50]
**89**	*A. fumigatus*	the marine fish *Mugil cephalus*	cytotoxic tests are in progress	[50]
**90**	*A. fumigatus*	the marine fish *Mugil cephalus*	cytotoxic tests are in progress	[50]
**91**	*A. fumigatus*	the marine fish *Mugil cephalus*	cytotoxic tests are in progress	[50]
**92**	*A.* sp. Z-4	the marine isopod *Ligia oceanica* collected in seaside of Dinghai in Zhoushan, Zhejiang Province of China	weak cytotoxic activity against PC3 cell line	[51]
**93**	*A.* sp. Z-4	the marine isopod *Ligia oceanica* collected in seaside of Dinghai in Zhoushan, Zhejiang Province of China	weak cytotoxic activity against PC3 cell line	[51]
**94**	*A.* sp. Z-4	the marine isopod *Ligia oceanica* collected in seaside of Dinghai, Zhejiang Province of China	moderate cytotoxic activities against PC3 and HCT116 cell lines	[52]
**95**	*A.* sp. Z-4	the marine isopod *Ligia oceanica* collected in seaside of Dinghai, Zhejiang Province of China	no biological activity was tested	[52]
**96**	*A.* sp. Z-4	the intestinal of the marine isopod *Ligia oceanica*	weak cytotoxic activities against PC3 cell line	[53]
**97**	*A.* sp. SCSIOW2	the deep marine sediment (2439 m) collected in the South China Sea	weak inhibitory activity on NO production induced by lipopolysaccharide (LPS)/INF-γ	[56]
**98**	*A. violaceofuscus*	the inner part of marine sponge *Reniochalina* sp. collected from Xisha Islands in South China Sea	nonsignificant anti-inflammatory activity against IL-10 expression of the LPS-induced THP-1 cells	[44]
**99**	*A. violaceofuscus*	the inner part of marine sponge *Reniochalina* sp. collected from Xisha Islands in South China Sea	potent anti-inflammatory activity against IL-10 expression of the LPS-induced THP-1 cells	[44]
**100**	*A. versicolor* ZLN-60	the mud (depth, 20 m) of the Yellow Sea, China	nonsignificant cytotoxic activities and lipid-lowering effect	[57]
	*A.* sp. BM-05 and BM-05ML	a brown algal species belonging to the genus *Sargassum* collected off Helgoland, North Sea, Germany	moderate cytotoxicities against K562, HCT116, A2780, and A2780CisR cell lines	[58]
**101**	*A. versicolor* ZLN-60	the mud (depth, 20 m) of the Yellow Sea, China	nonsignificant cytotoxic activities and lipid-lowering effect	[57]
**102**	*A. versicolor* ZLN-60	the mud (depth, 20 m) of the Yellow Sea, China	potent lipid-lowering effect; nonsignificant cytotoxic activities	[57]
**103**	*A. versicolor* ZLN-60	the mud (depth, 20 m) of the Yellow Sea, China	nonsignificant cytotoxic activities and lipid-lowering effect	[57]
**104**	*A. versicolor* ZLN-60	the mud (depth, 20 m) of the Yellow Sea, China	nonsignificant cytotoxic activities and lipid-lowering effect	[57]
**105**	*A. terreus* SCSGAF0162	the gorgonian coral *Echinogorgia aurantiaca* in the South China Sea	nonsignificant antifouling activity towards larvae of the barnacle *B. amphitrite*	[59]
**106**	*A.* sp. SCSIO 41501	the gorgonian *Melitodes squamata* collected from the South China Sea, Sanya, China	moderate antiviral activity against HSV-1 under non-cytotoxic concentrations against Vero cells	[23]
**107**	*A. similanensis* KUFA 0013	the marine sponge *Rhabdermia* sp. collected in coral reef of Similan Islands, Phang Nga, Thailand	nonsignificant cytotoxic and antibacterial activities	[60]
**108**	*A. versicolor* TA01-14	a gorgonian *Carijoa* sp. GX-WZ-2010001 collected in Weizhou coral reefs in the South China Sea	weak cytotoxic activity; nonsignificant brine shrimp lethality, antibacterial and antiviral activities, as well as AChE, Top I, and α-glucosacharase inhibition	[61]
**109**	*A. similanensis* KUFA 0013	the marine sponge *Rhabdermia* sp. collected in coral reef of Similan Islands, Phang Nga, Thailand	weak cytotoxicity; nonsignificant antibacterial activities against four reference strains	[60]
**110**	*A. niger* SCSIO Jcsw6F30	a marine alga *Sargassum* sp. collected in Yongxing Island, South China Sea	potent cytotoxic activity against TZM-bl cells; moderate anti-HIV-1 activity against HIV-1 SF162	[62]
**111**	*A. niger* SCSIO Jcsw6F30	a marine alga *Sargassum* sp. collected in Yongxing Island, South China Sea	no biological activity was tested	[62]
**112**	*A.* sp. XS20090B15	the *Muricella abnormaliz* gorgonian collected from the Xisha Islands coral reef in South China Sea	nonsignificant antiviral activity against RSV virus-induced cytopathogenicity in Hep-2 cells	[63]
**113**	*A.* sp. XS20090B15	the *Muricella abnormaliz* gorgonian collected from the Xisha Islands coral reef in South China Sea	potent antiviral activity against RSV virus-induced cytopathogenicity in Hep-2 cells	[63]
**114**	*A. versicolor* A-21-2-7	the deep-sea sediment (3002 m) in South China Sea	no biological activity was tested	[64]
**115**	*A. flavus* KMM 4650	Sakhalin Bay marine sediments (32 m, Sea of Okhotsk)	nonsignificant antimicrobial activity	[65]
**116**	*A. versicolor*	the inner part of gorgonian *D. gemmacea* collected from the Xisha Islands coral reef of the South China Sea	moderate antibacterial activities and brine shrimp lethality; nonsignificant cytotoxicities	[66]
**117**	*A. versicolor*	the inner part of gorgonian *D. gemmacea* collected from the Xisha Islands coral reef of the South China Sea	moderate antibacterial activities and brine shrimp lethality; nonsignificant cytotoxicities	[66]
**118**	*A. ochraceus* LCJ11-102	the gorgonian *Dichotella gemmacea* (Valenciennes) collected in Lingao, Hainan province of China	moderate antimicrobial activity against *E. aerogenes*; nonsignificant cytotoxic activities	[67]
**119**	*A. ochraceus* LCJ11-102	the gorgonian *Dichotella gemmacea* (Valenciennes) collected in Lingao, Hainan province of China	nonsignificant antimicrobial and cytotoxic activities	[67]
**120**	*A. ochraceus* LCJ11-102	the gorgonian *Dichotella gemmacea* (Valenciennes) collected in Lingao, Hainan province of China	nonsignificant antimicrobial and cytotoxic activities	[67]
**121**	*A. ochraceus* LCJ11-102	the gorgonian *Dichotella gemmacea* (Valenciennes) collected in Lingao, Hainan province of China	moderate antimicrobial activity against *E. aerogenes*; nonsignificant cytotoxic activities	[67]
**122**	*A. ochraceus* LCJ11-102	the gorgonian *Dichotella gemmacea* (Valenciennes) collected in Lingao, Hainan province of China	nonsignificant antimicrobial and cytotoxic activities	[67]
**123**	*A. sclerotiorum* and *P. citrinum*	the gorgonian *Muricella flexuosa* collected from the South China Sea, Sanya, Hainan Province, China	potent brine shrimp lethality and cytotoxic activities; nonsignificant antibacterial and anti-biofilm activities	[49]
**124**	*A. sclerotiorum* and *P. citrinum*	the gorgonian *Muricella flexuosa* collected from the South China Sea, Sanya, Hainan Province, China	moderate brine shrimp lethality and cytotoxic activities; nonsignificant antibacterial and anti-biofilm activities	[49]
**125**	*A*. sp. (33241)	the mangrove *Bruguiera sexangula* var. *rhynchopetala* collected in the South China Sea	nonsignificant antibacterial and cytotoxic activities	[68]
**126**	*A. terreus* FA009	the marine sediment collected in Jeju Island, Korea	moderate enhancement effect on insulin sensitivity	[69]
**127**	*A. terreus* FA009	the marine sediment collected in Jeju Island, Korea	moderate enhancement effect on insulin sensitivity	[69]
**128**	*A. sclerotiorum* and *P. citrinum*	the gorgonian *Muricella flexuosa* collected from the South China Sea, Sanya, Hainan Province, China	weak brine shrimp lethality; nonsignificant cytotoxic, antibacterial and anti-biofilm activities	[49]
**129**	*A. sydowii* SP-1	the marine sediment sample collected from site in the Antarctic Great Wall Station	weak antimicrobial activities against MRSA and MRSE	[70]
**130**	*A.* sp. WU 243	the crab *Xenograpsus testudinatus* collected from a Kueishantao hydrothermal vent, Taiwan, China	no biological activity was tested	[71]

## Supplementary Materials

The following are available online at https://www.mdpi.com/1660-3397/18/1/54/s1, the structures of all 398 new metabolites produced by marine-derived *Aspergillus* species from 2014 to 2018, as well as references.
