# Supplementary material for: Recent Discovery of Heterocyclic Alkaloids from Marine-Derived Aspergillus Species
Source: Mar Drugs. 2020 Jan 14;18(1):54. doi: 10.3390/md18010054 (PMC7024353; doi:10.3390/md18010054)
Supplement: Supplementary file 1 [file marinedrugs-18-00054-s001.pdf]

Recent Discovery of Heterocyclic Alkaloids from Marine-Derived *Aspergillus* Species

Kuo Xu [1], Xiaolong Yuan [1], Chen Li [2,3] and Xiao-Dong Li [2, 3*]

1 Tobacco Research Institute of Chinese Academy of Agricultural Sciences, Qingdao 266101, People's Republic of China; xukuoworld@126.com (K.X.); yuanxiaolong@caas.cn (X.-L.Y.)

2 Yantai Institute of Coastal Zone Research, Chinese Academy of Sciences, Yantai 264003, China; Lychees6601@163.com (C.L.); imnli@163.com (X.-D.L.)

3 Key Laboratory of Marine Biotechnology in Universities of Shandong (Ludong University), School of Life Sciences, Ludong University, Yantai 264025, China; Lychees6601@163.com (C.L.); imnli@163.com (X.-D.L.)

* Corresponding authors: imnli@163.com (X.-D.L.), Tel.: +86-535-2109018 (X.-D.L.)

1

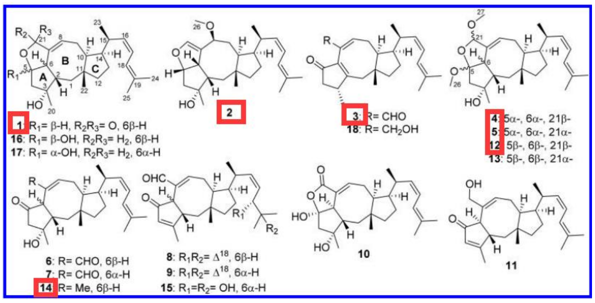

2

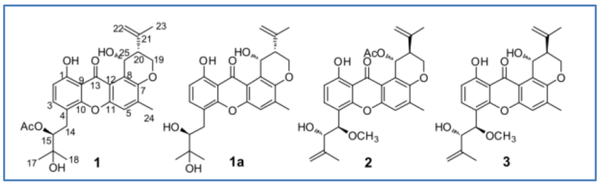

3

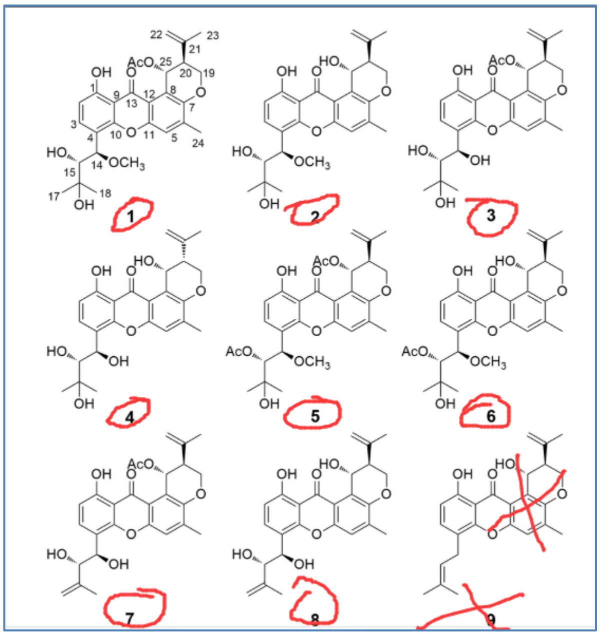

4

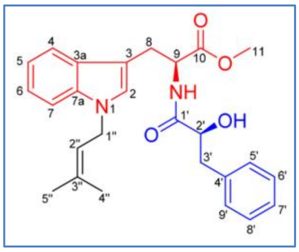

5

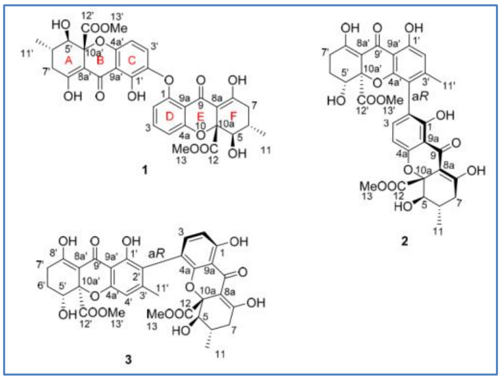

6

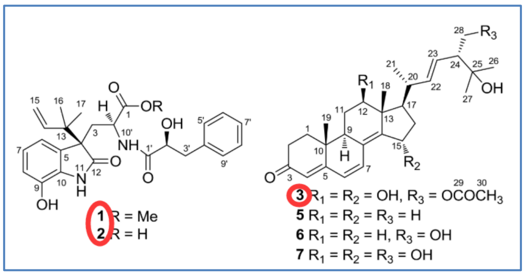

7

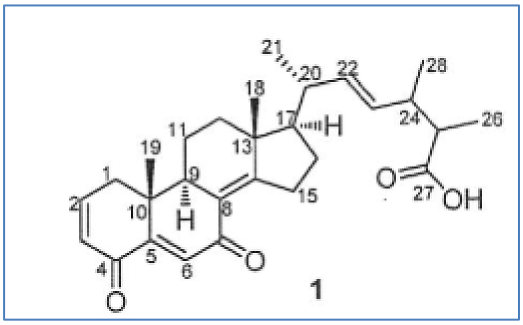

8

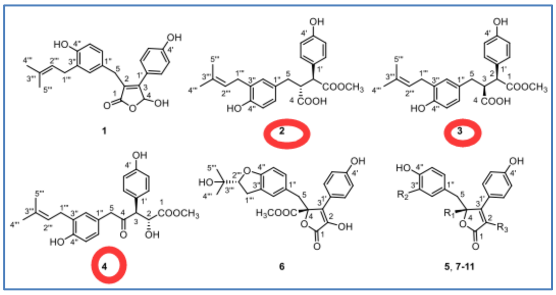

9

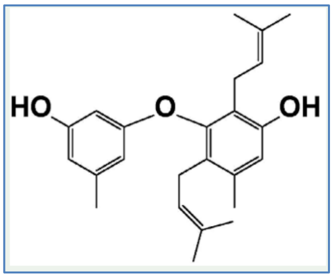

10

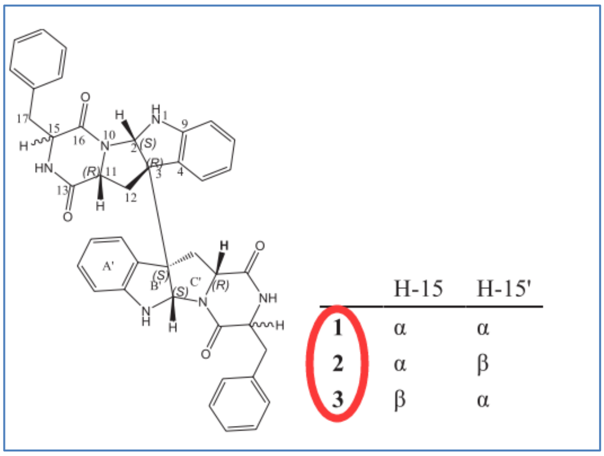

11

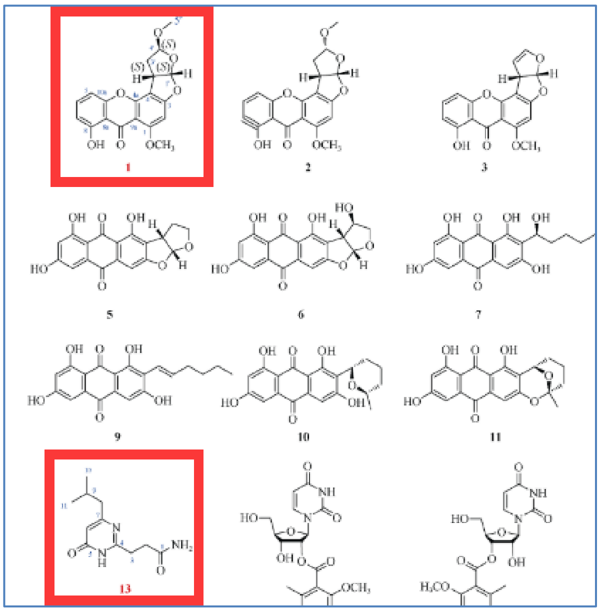

12

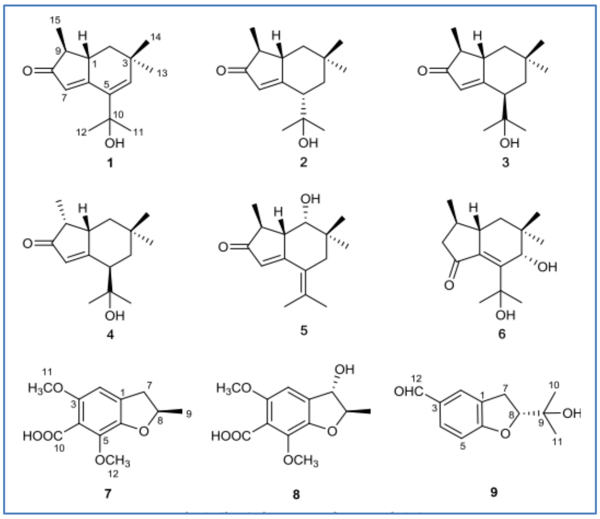

13

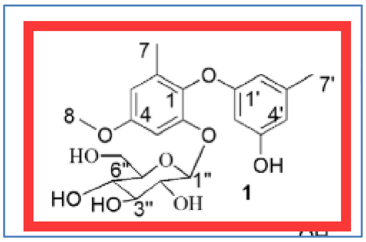

14

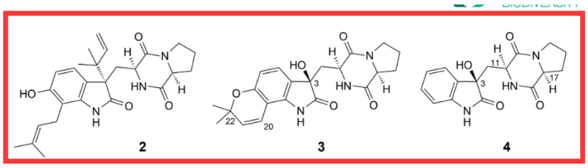

15

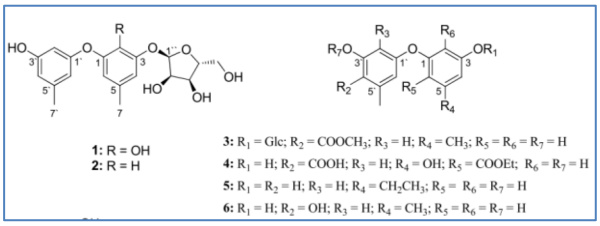

16

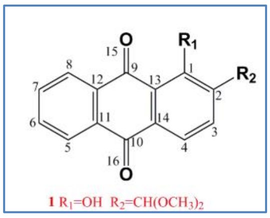

17

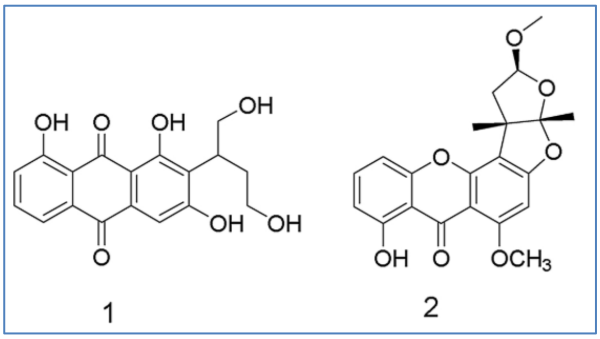

18

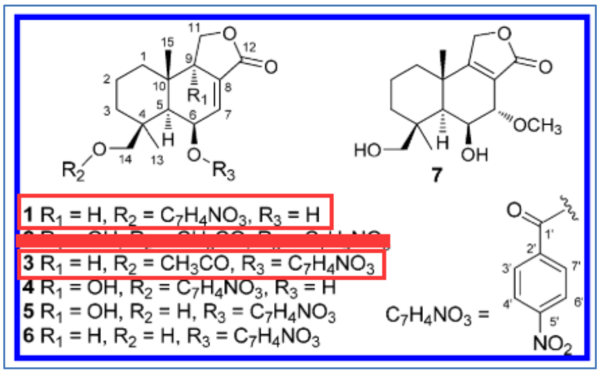

19

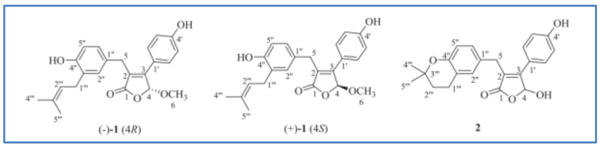

20

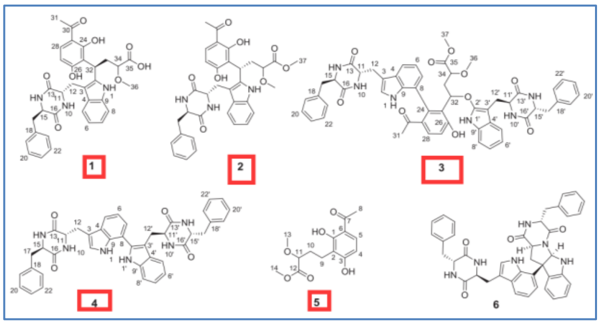

21

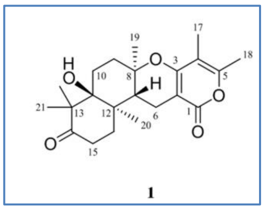

22

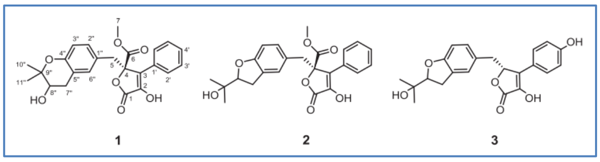

23

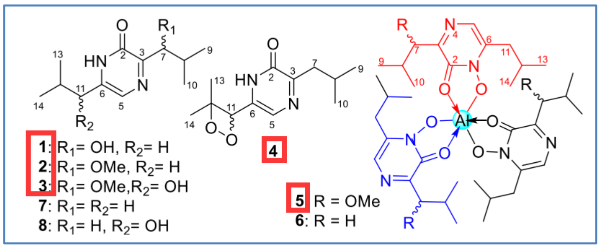

24

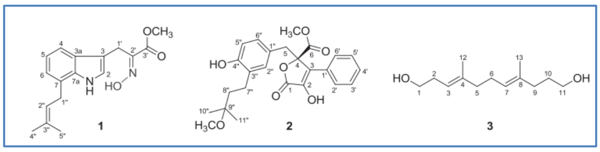

25

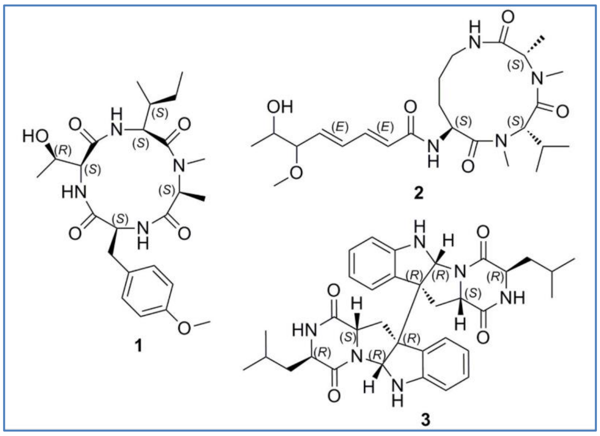

26

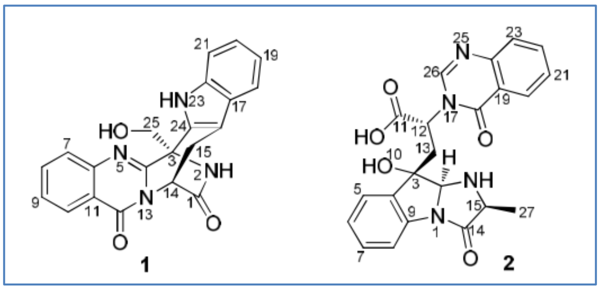

27

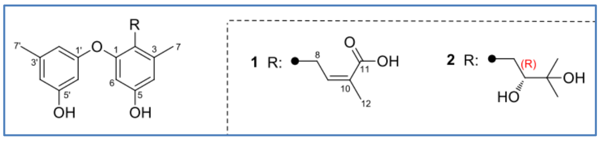

28

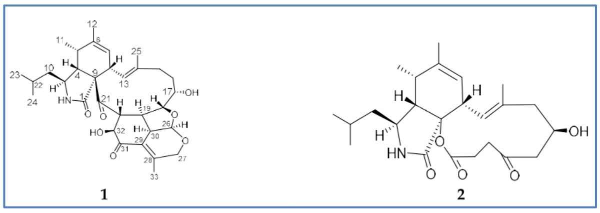

29

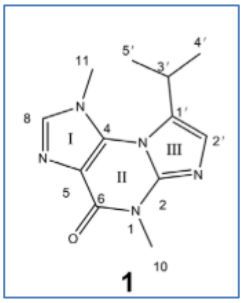

30

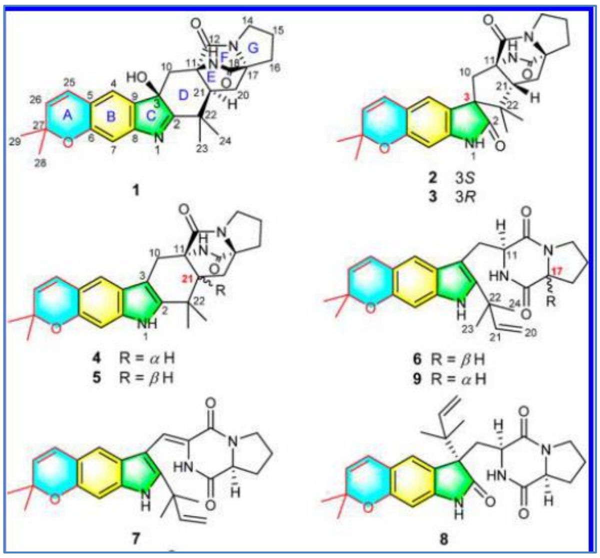

31

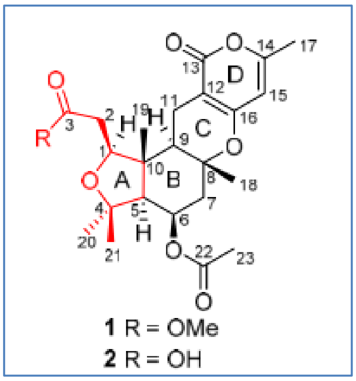

32

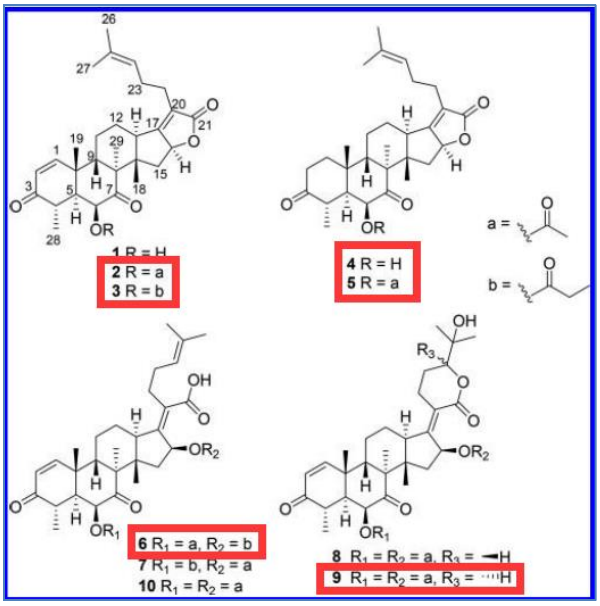

33

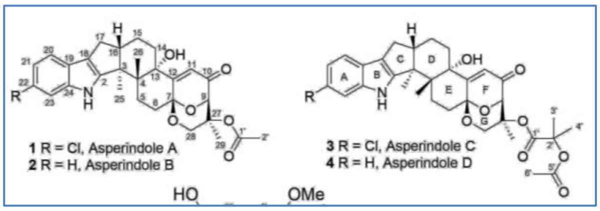

34

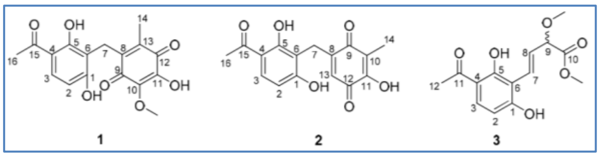

35

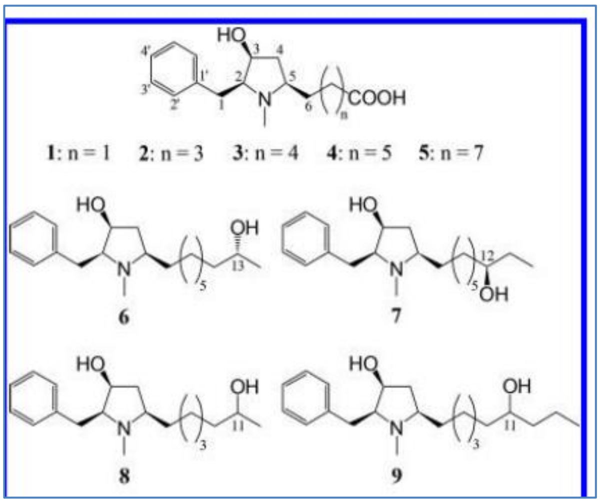

36

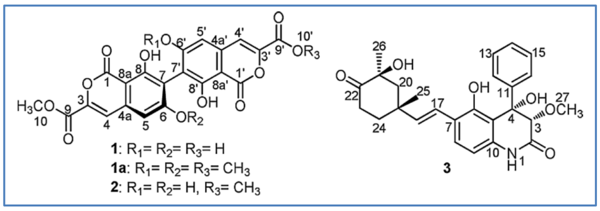

37

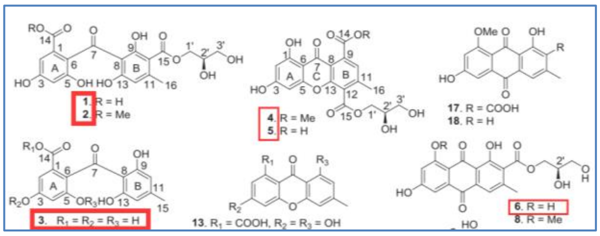

38

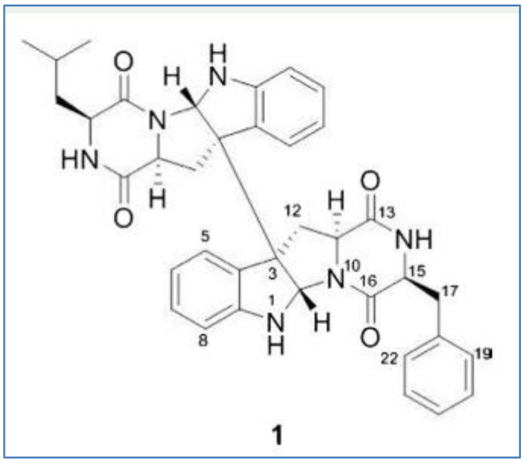

39

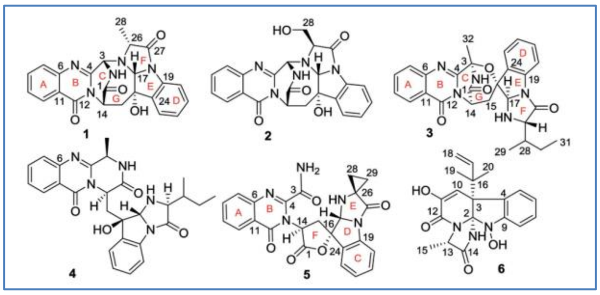

40

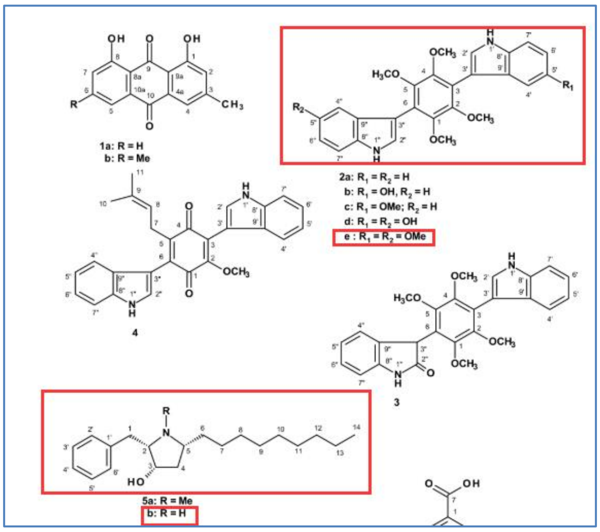

41

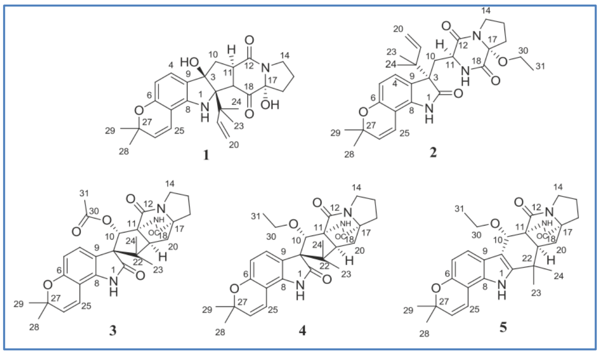

42

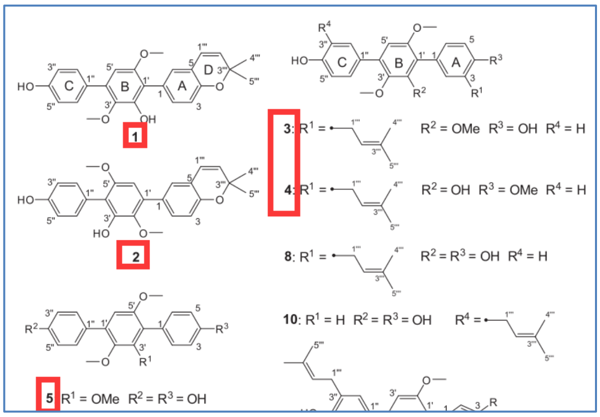

43

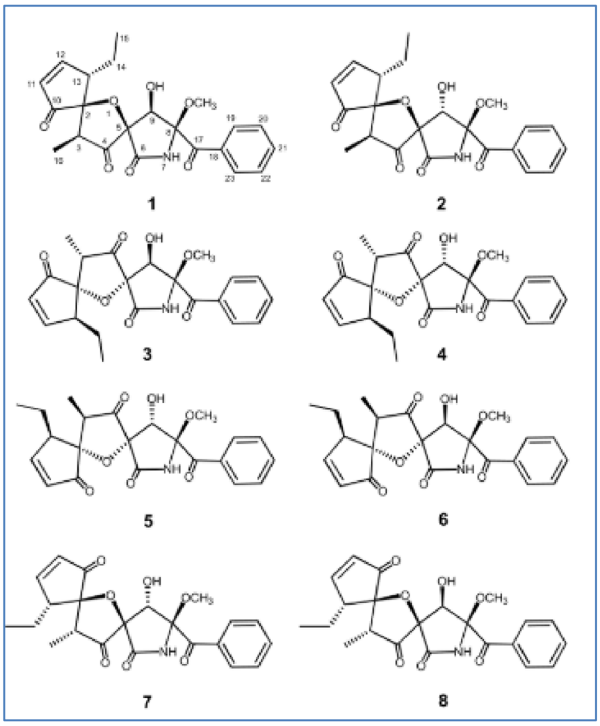

44

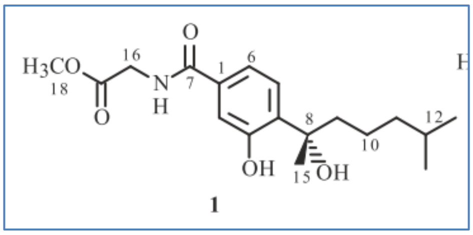

45

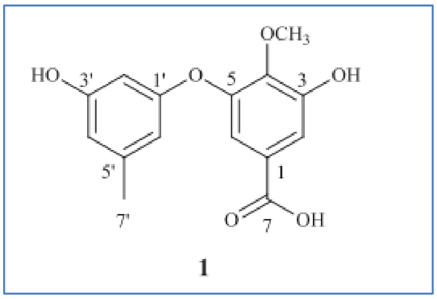

46

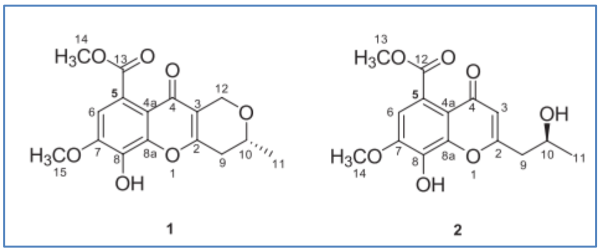

47

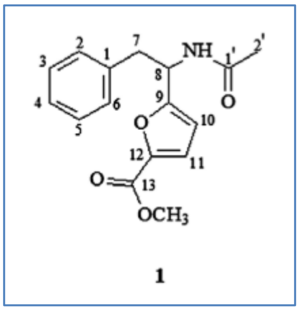

48

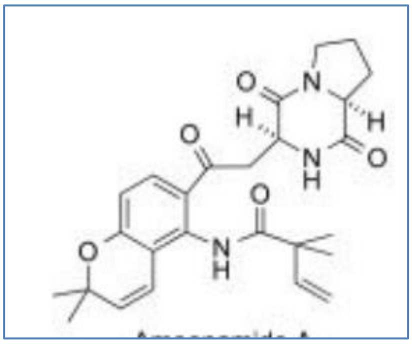

49

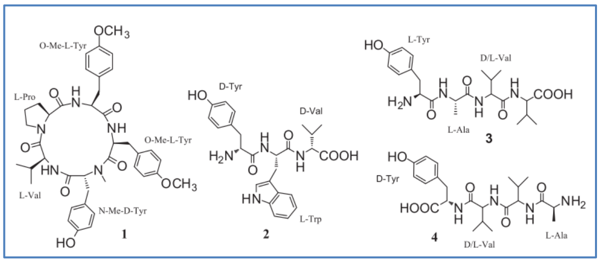

50

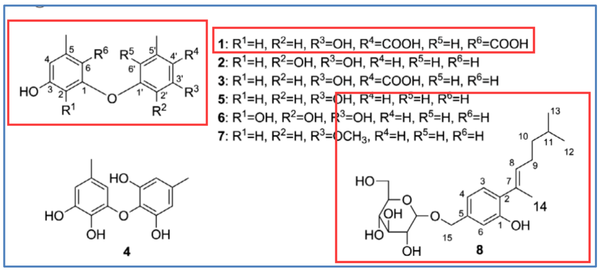

51

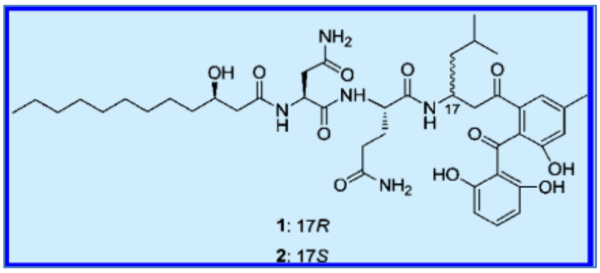

52

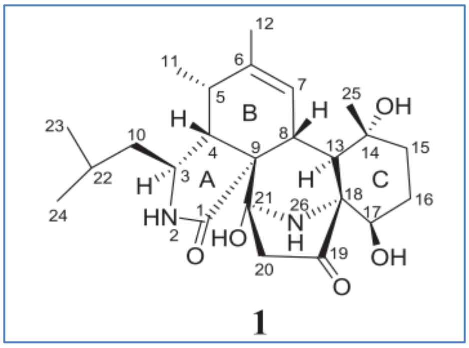

53

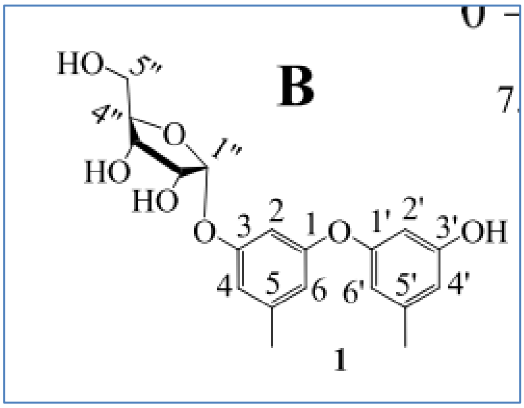

54

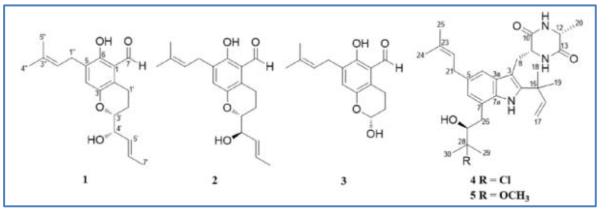

55

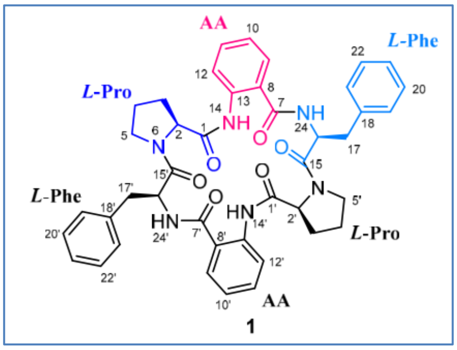

56

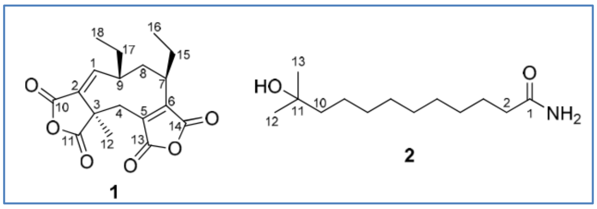

57

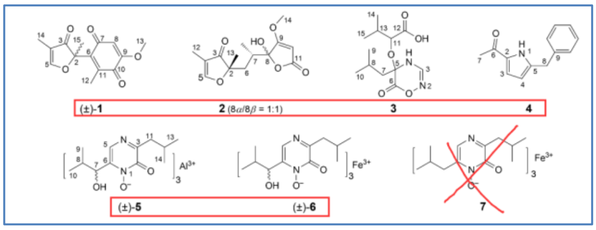

58

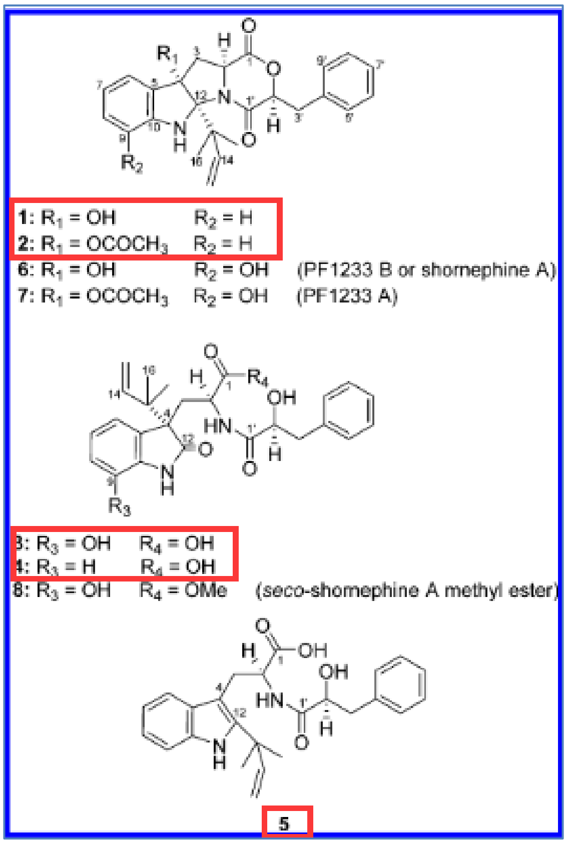

59

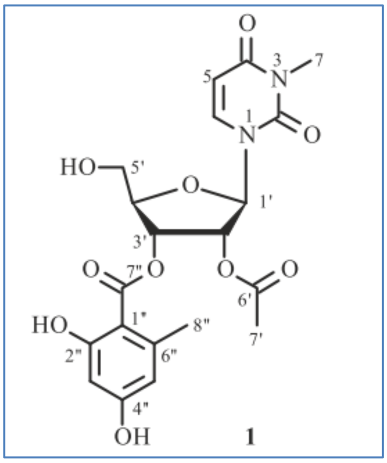

60

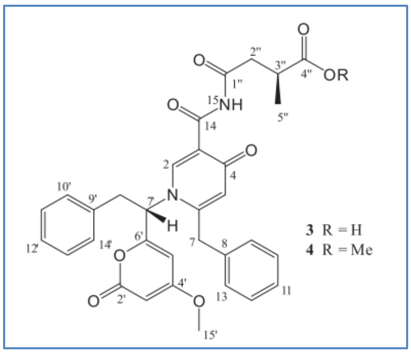

61

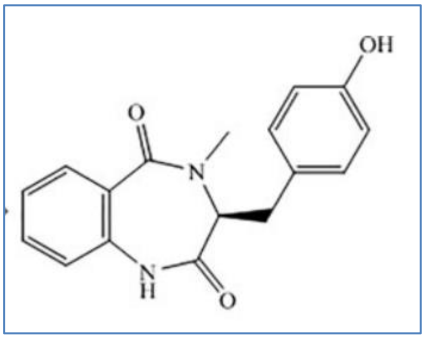

62

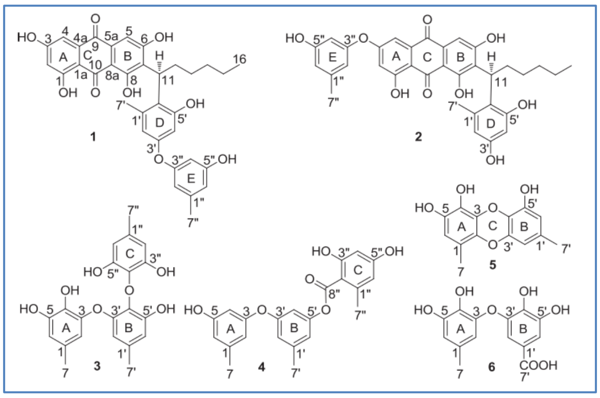

63

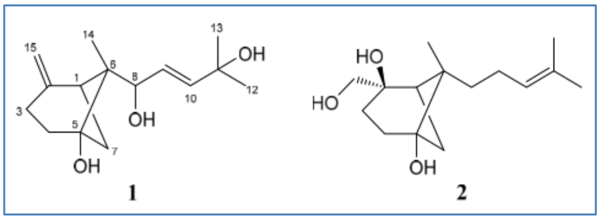

64

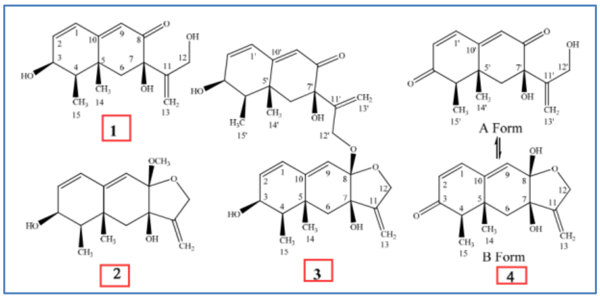

65

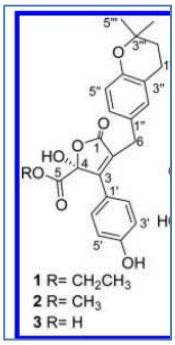

66

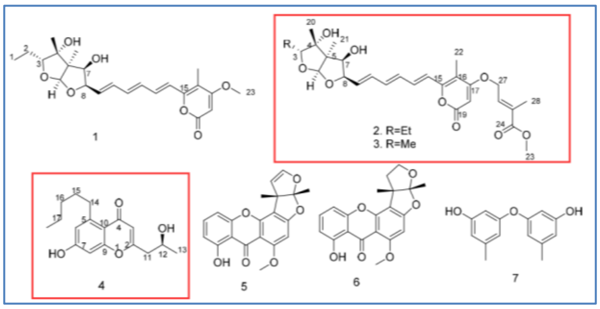

67

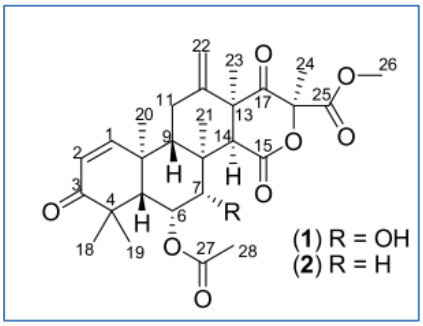

68

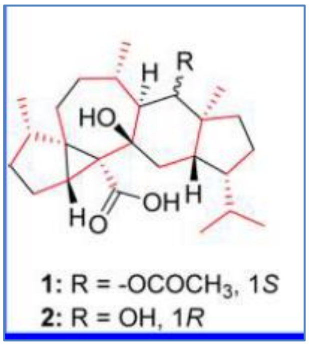

69

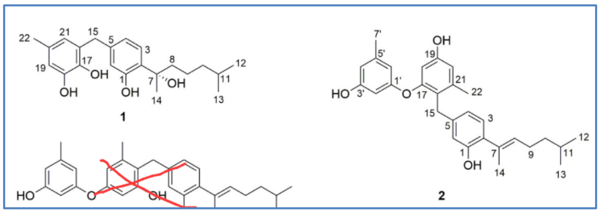

70

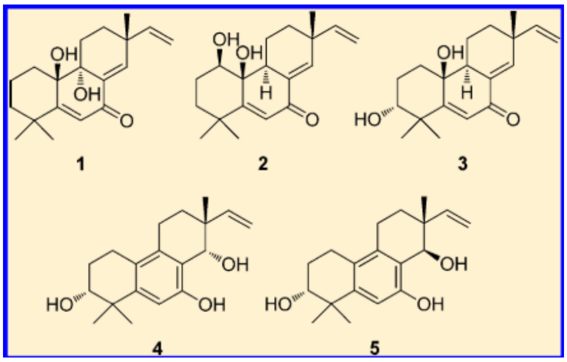

71

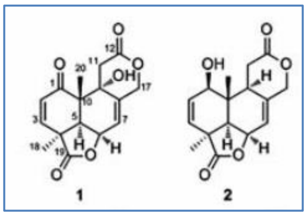

72

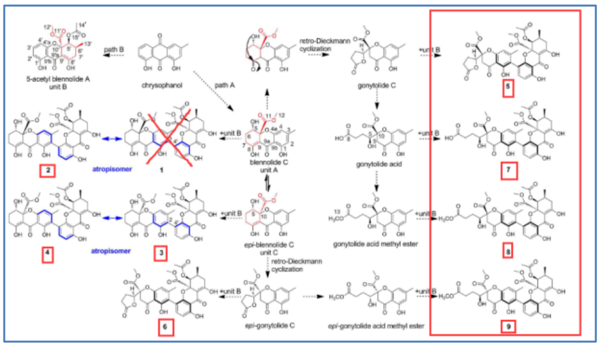

73

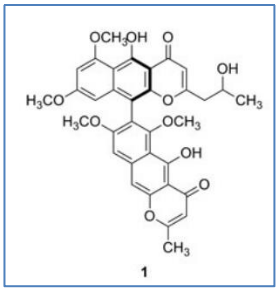

74

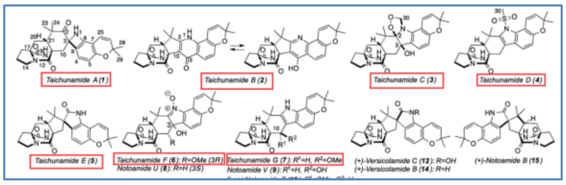

75

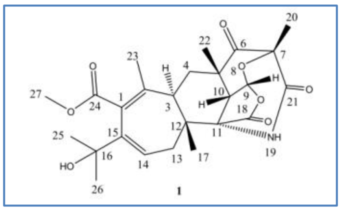

76

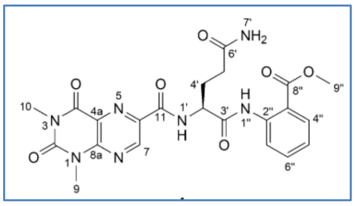

77

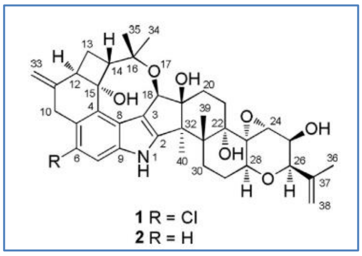

78

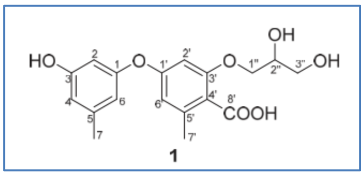

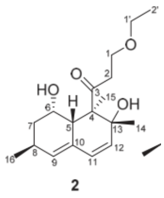

79

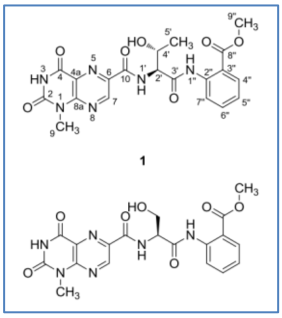

80

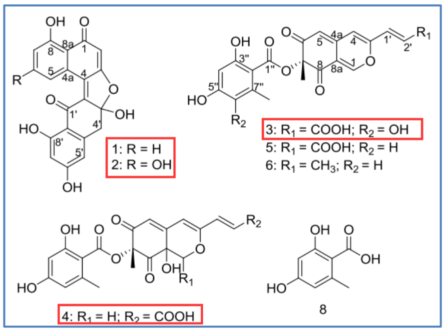

81

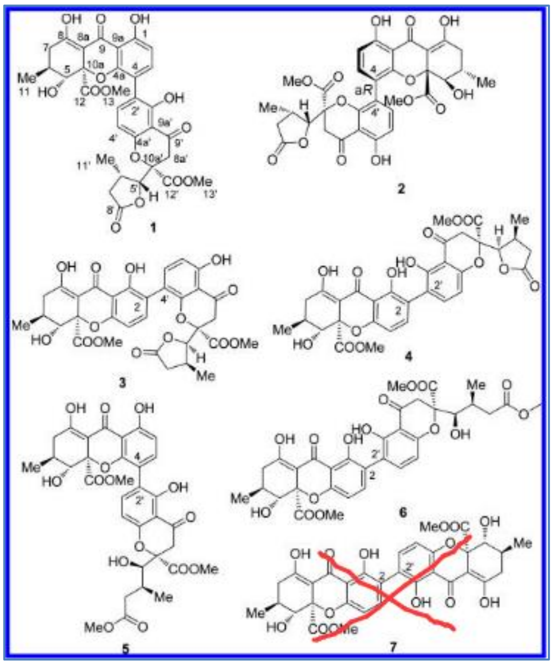

82

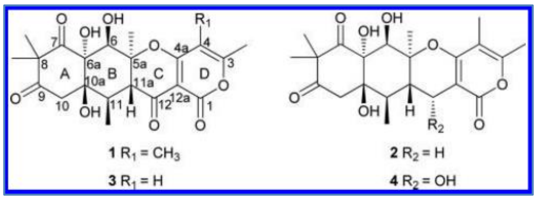

83

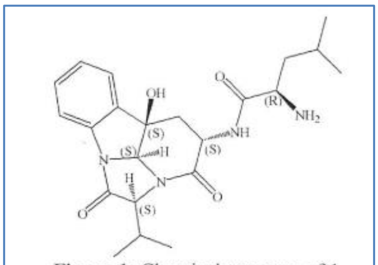

Figure 1: Chemical structure of **1**.

84

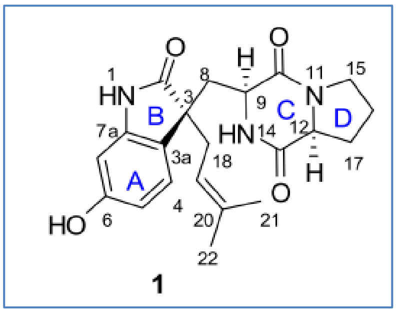

85

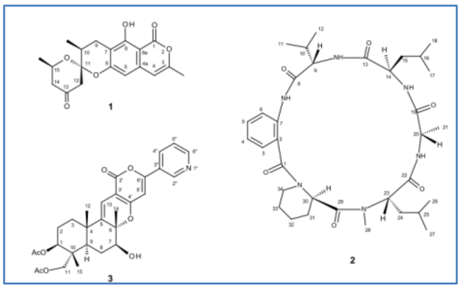

86

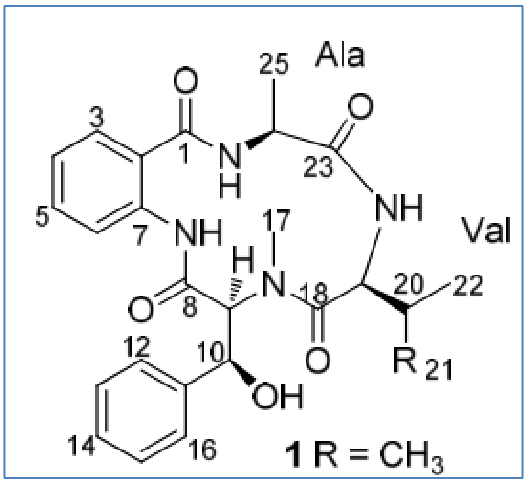

87

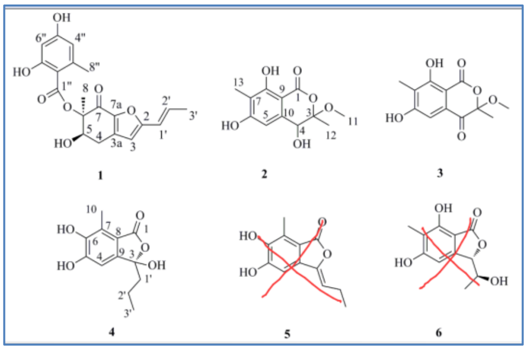

88

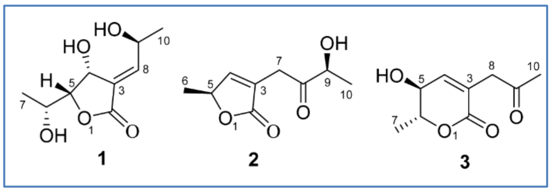

89

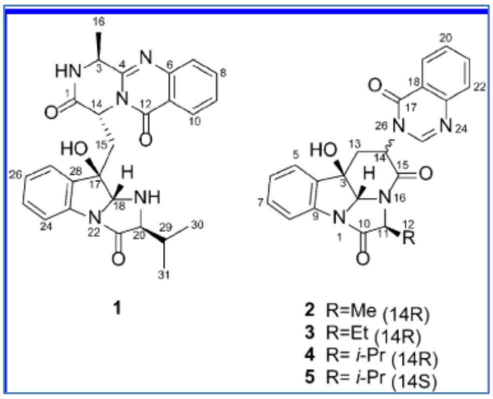

92

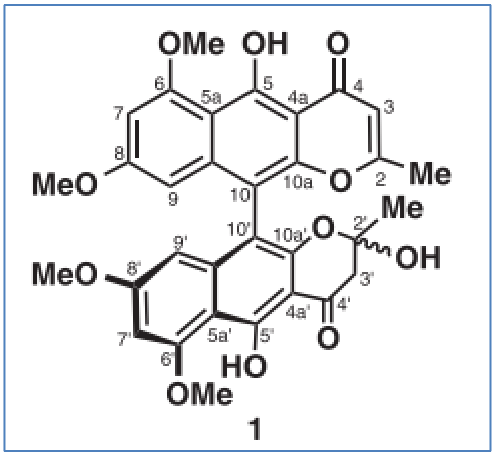

90

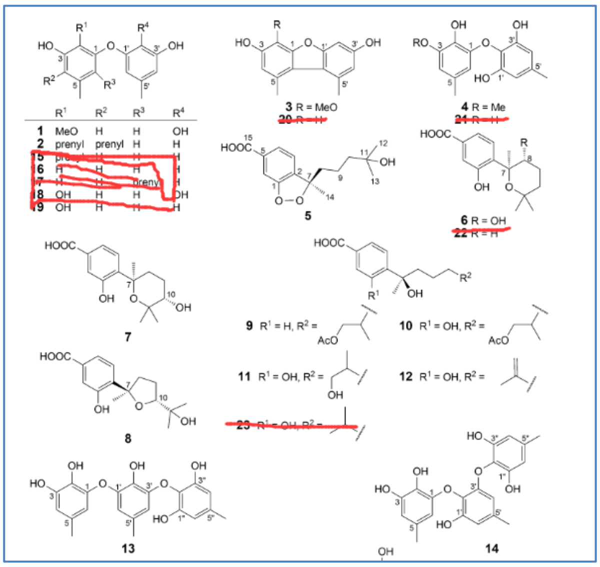

93

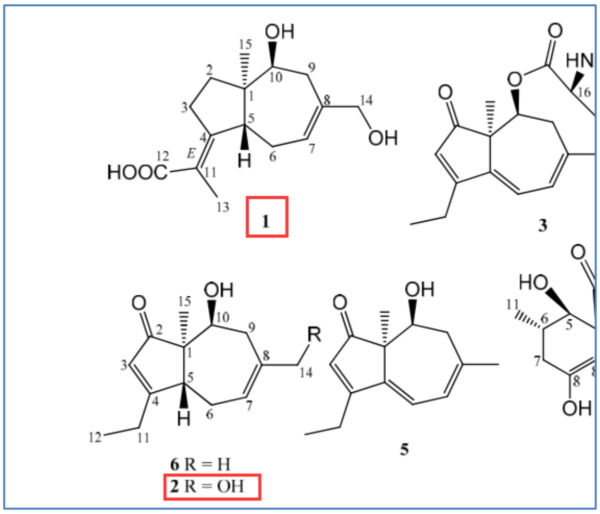

91

94

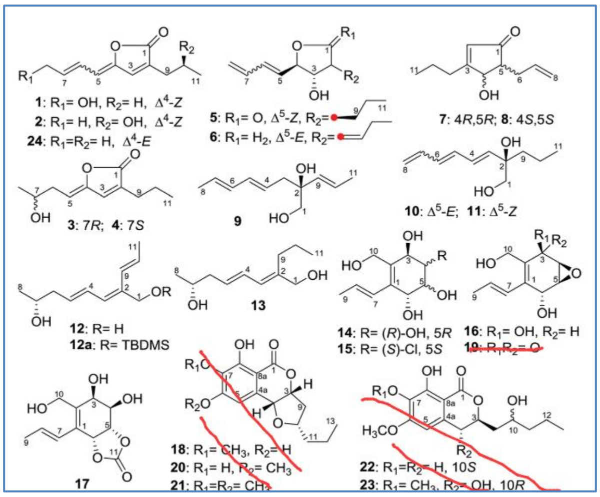

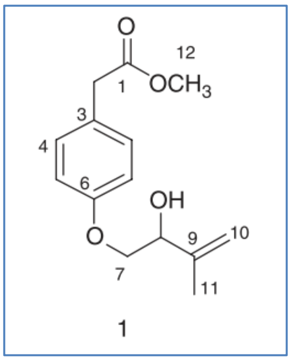

95

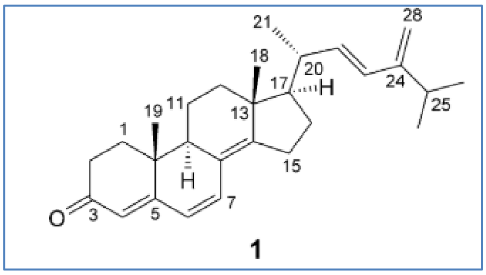

96

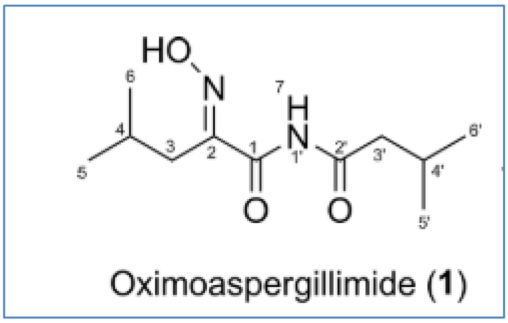

97

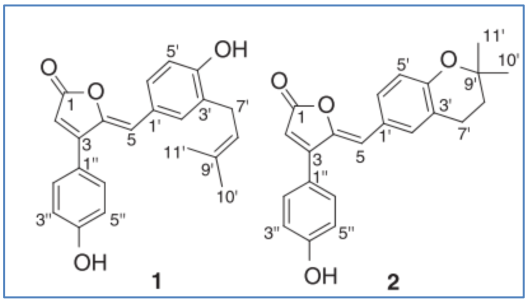

98

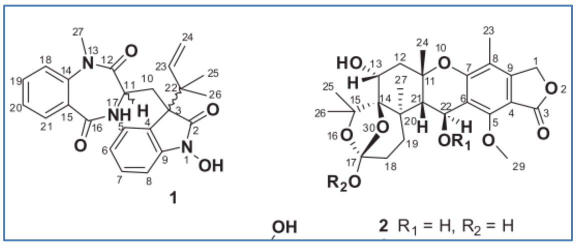

99

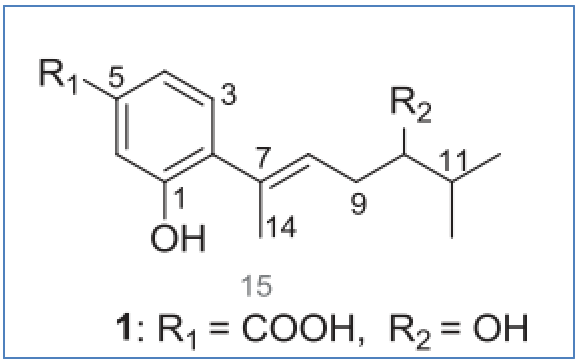

100

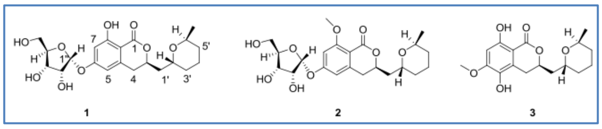

101

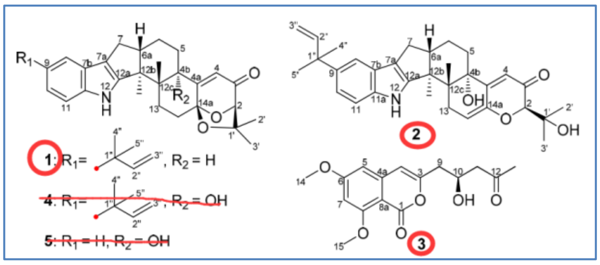

102

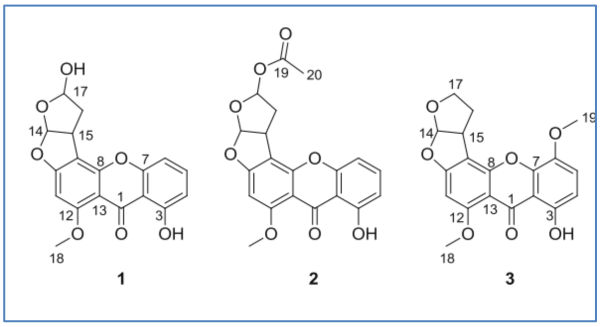

103

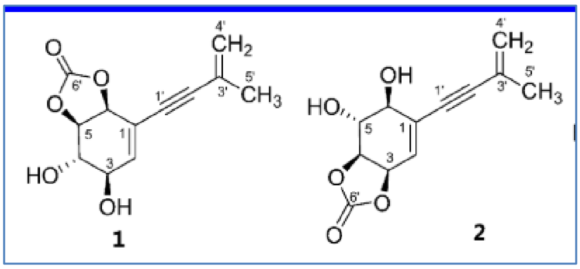

104

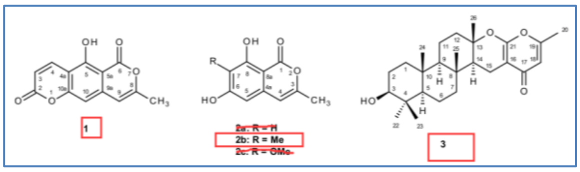

105

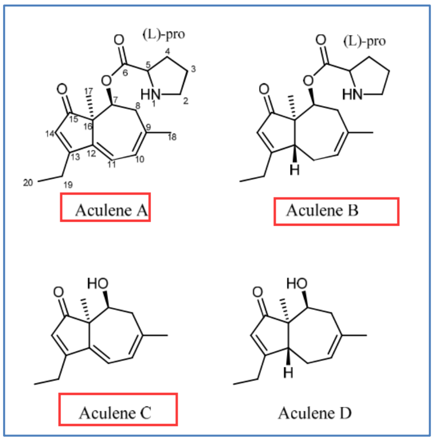

106

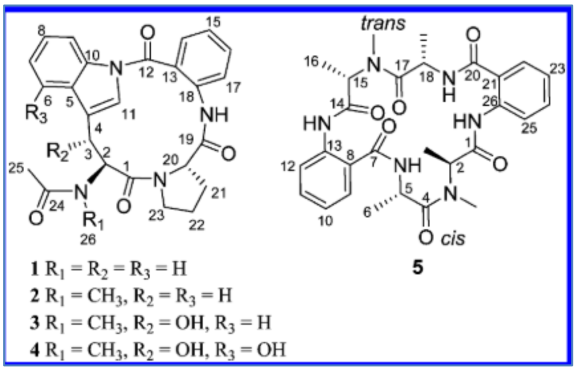

107

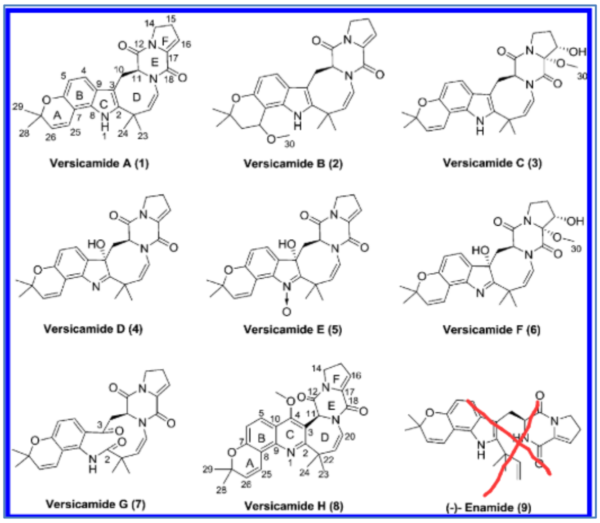

108

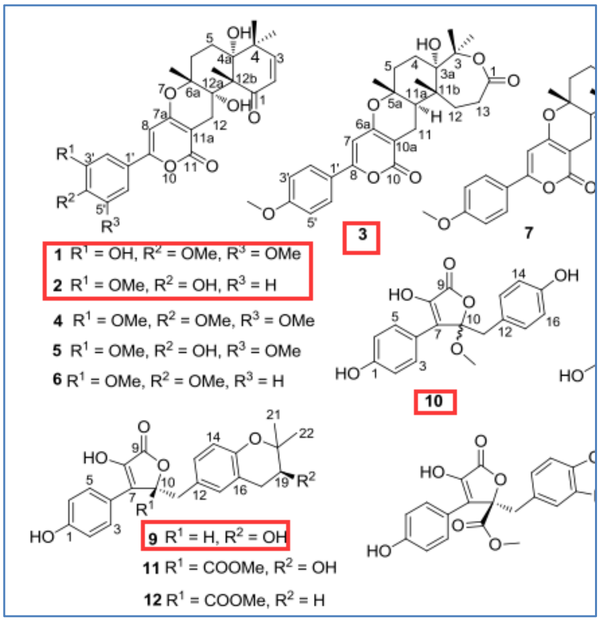

109

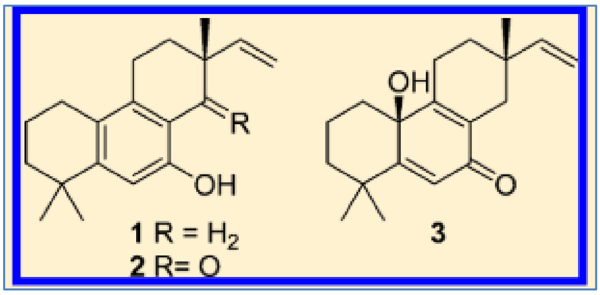

110

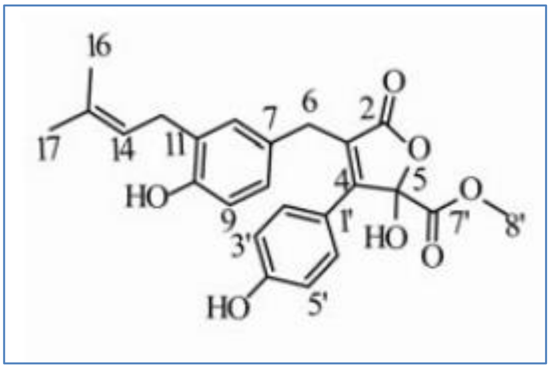

111

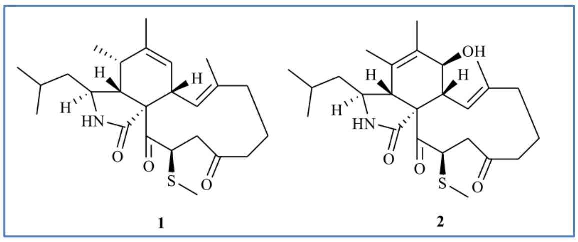

112

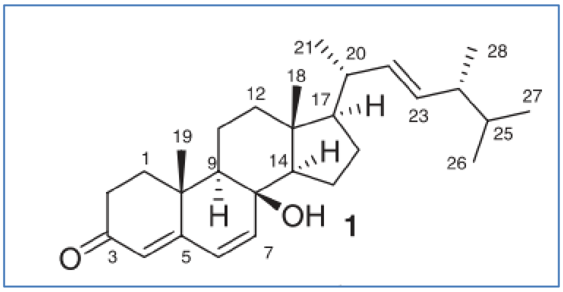

113

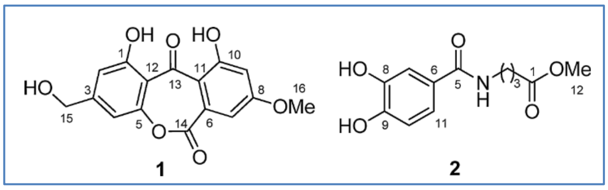

114

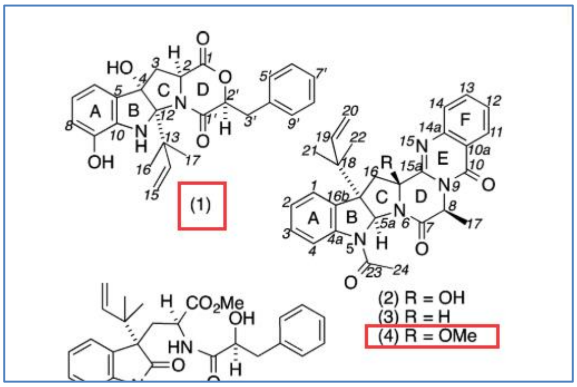

115

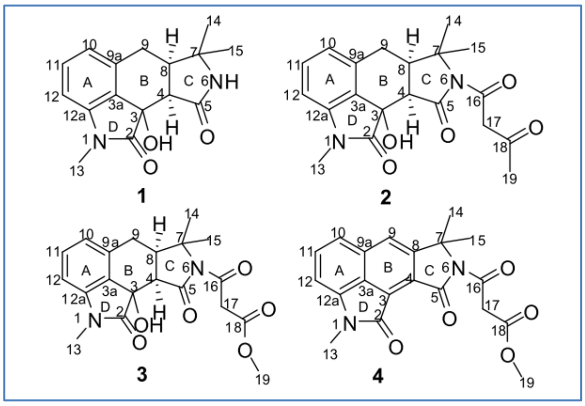

116

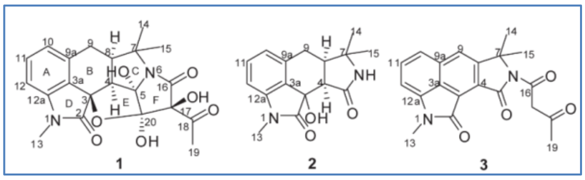

117

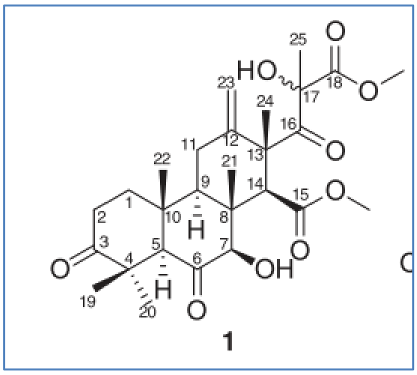

118

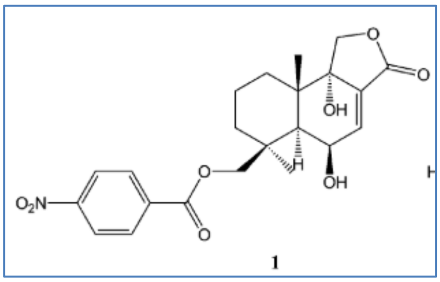

119

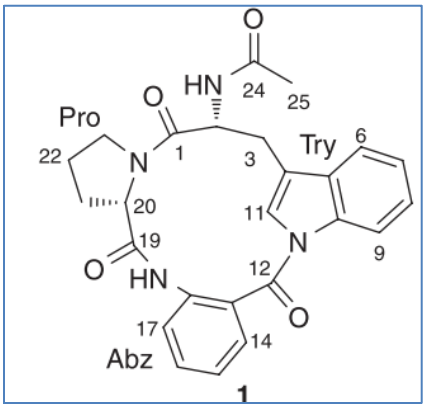

120

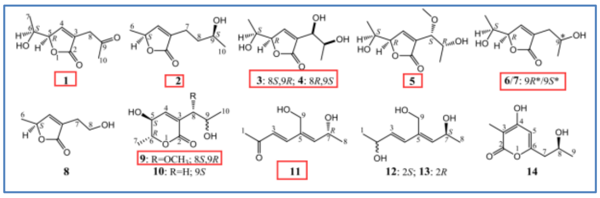

121

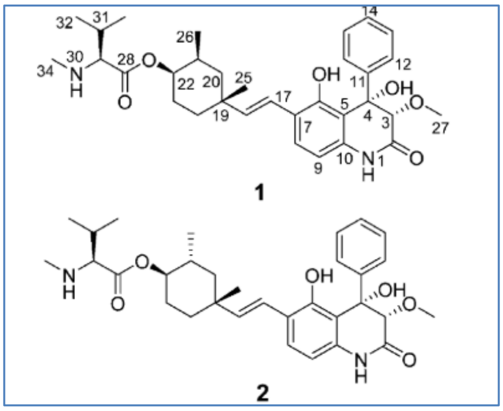

122

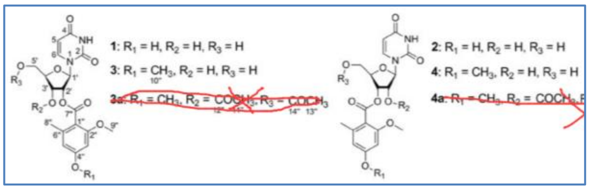

123

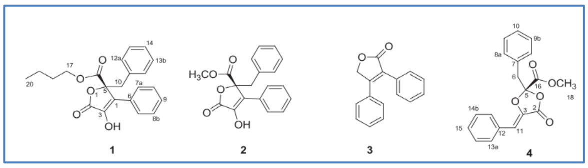

References:

1. Zhu, T. H.; Lu, Z. Y.; Fan, J.; Wang, L. P.; Zhu, G. L.; Wang, Y.; Li, X.; Hong, K.; Piyachaturawat, P.; Chairoungdua, A.; Zhu, W. M., Ophiobolins from the Mangrove Fungus Aspergillus ustus. *Journal of Natural Products* **2018,** *81* (1), 2-9.

2. Zhu, A.; Zhang, X. W.; Zhang, M.; Li, W.; Ma, Z. Y.; Zhu, H. J.; Cao, F., Aspergixanthones I-K, New Anti-Vibrio Prenylxanthones from the Marine-Derived Fungus Aspergillus sp ZA-01. *Marine Drugs* **2018,** *16* (9).

3. Zhu, A.; Yang, M. Y.; Zhang, Y. H.; Shao, C. L.; Wang, C. Y.; Hu, L. D.; Cao, F.; Zhu, H. J., Absolute Configurations of 14,15-Hydroxylated Prenylxanthones from a Marine-Derived Aspergillus sp Fungus by Chiroptical Methods. *Scientific Reports* **2018,** *8*.

4. Zhou, R.; Liao, X. J.; Li, H. B.; Li, J.; Peng, P. J.; Zhao, B. X.; Xu, S. H., Isolation and Synthesis of Misszrtine A: A Novel Indole Alkaloid From Marine Sponge-Associated Aspergillus sp SCSIO XWS03F03. *Frontiers in Chemistry* **2018,** *6*.

5. Yu, G. H.; Wu, G. W.; Sun, Z. C.; Zhang, X. M.; Che, Q.; Gu, Q. Q.; Zhu, T. J.; Li, D. H.; Zhang, G. J., Cytotoxic Tetrahydroxanthone Dimers from the Mangrove-Associated Fungus Aspergillus versicolor HDN1009. *Marine Drugs* **2018,** *16* (9).

6. Yang, S. Q.; Li, X. M.; Li, X.; Chi, L. P.; Wang, B. G., Two New Diketomorpholine Derivatives and a New Highly Conjugated Ergostane-Type Steroid from the Marine Algal-Derived Endophytic Fungus Aspergillus alabamensis EN-547. *Marine Drugs* **2018,** *16* (4).

7. Yang, M. Y.; Yang, J. K.; Yang, J. K.; Hu, L. D.; Zhu, H. J.; Cao, F., New Oxygenated Steroid from the Marine-Derived Fungus Aspergillus flavus. *Natural Product Communications* **2018,** *13* (8), 949-951.

8. Yang, L. H.; Ou-Yang, H.; Yan, X.; Tang, B. W.; Fang, M. J.; Wu, Z.; Chen, J. W.; Qiu, Y. K., Open-Ring Butenolides from a Marine-Derived Anti-Neuroinflammatory Fungus Aspergillus terreus Y10. *Marine Drugs* **2018,** *16* (11).

9. Xu, X. L.; Yang, H. J.; Xu, H. T.; Yin, L. Y.; Chen, Z. K.; Shen, H. H., Diphenyl ethers from a marine-derived isolate of Aspergillus sp CUGB-F046. *Natural Product Research* **2018,** *32* (7), 821-825.

10. Xu, J. Z.; Hu, Q.; Ding, W. J.; Wang, P. M.; Di, Y. N., New asymmetrical bispyrrolidinoindoline diketopiperazines from the marine fungus Aspergillus sp DX4H. *Natural Product Research* **2018,** *32* (7), 815-820.

11. Wu, Z. H.; Liu, D.; Xu, Y.; Chen, J. L.; Lin, W. H., Antioxidant xanthones and anthraquinones isolated from a marine-derived fungus Aspergillus versicolor. *Chinese Journal of Natural Medicines* **2018,** *16* (3), 219-224.

12. Wu, Z. D.; Li, D. Y.; Zeng, F. R.; Tong, Q. Y.; Zheng, Y. Y.; Liu, J. J.; Zhou, Q.; Li, X. N.; Chen, C. M.; Lai, Y. J.; Zhu, H. C.; Zhang, Y. H., Brasilane sesquiterpenoids and dihydrobenzofuran derivatives from Aspergillus terreus CFCC 81836. *Phytochemistry* **2018,** *156*, 159-166.

13. Wu, X. M.; Chen, Z.; Ding, W. J.; Liu, Y.; Ma, Z. J., Chemical constituents of the fermentative extracts of marine fungi Phoma sp. CZD-F11 and Aspergillus sp. CZD-F18 from Zhoushan Archipelago, China. *Natural Product Research* **2018,** *32* (13), 1562-1566.

14. Wen, H. L.; Liu, X. R.; Zhang, Q.; Deng, Y. F.; Zang, Y.; Wang, J. P.; Liu, J. J.; Zhou, Q.;

Hu, L. Z.; Zhu, H. C.; Chen, C. M.; Zhang, Y. H., Three New Indole Diketopiperazine Alkaloids from Aspergillus ochraceus. *Chemistry & Biodiversity* **2018,** *15* (4).

15. Wang, Y. N.; Mou, Y. H.; Dong, Y.; Wu, Y.; Liu, B. Y.; Bai, J.; Yan, D. J.; Zhang, L.; Feng, D. Q.; Pei, Y. H.; Hu, Y. C., Diphenyl Ethers from a Marine-Derived Aspergillus sydowii. *Marine Drugs* **2018,** *16* (11).

16. Wang, W. Y.; Chen, R. X.; Luo, Z. H.; Wang, W.; Chen, J. M., Antimicrobial activity and molecular docking studies of a novel anthraquinone from a marine-derived fungus Aspergillus versicolor. *Natural Product Research* **2018,** *32* (5), 558-563.

17. Tian, Y. Q.; Lin, S. T.; Kumaravel, K.; Zhou, H.; Wang, S. Y.; Liu, Y. H., Polyketide-derived metabolites from the sponge-derived fungus Aspergillus sp F40. *Phytochemistry Letters* **2018,** *27*, 74-77.

18. Tan, Y. H.; Yang, B.; Lin, X. P.; Luo, X. W.; Pang, X. Y.; Tang, L.; Liu, Y. H.; Li, X. J.; Zhou, X. F., Nitrobenzoyl Sesquiterpenoids with Cytotoxic Activities from a Marine-Derived Aspergillus ochraceus Fungus. *Journal of Natural Products* **2018,** *81* (1), 92-97.

19. Sun, Y. T.; Liu, J. T.; Li, L.; Gong, C.; Wang, S. P.; Yang, F.; Hua, H. M.; Lin, H. W., New butenolide derivatives from the marine sponge-derived fungus Aspergillus terreus. *Bioorganic & Medicinal Chemistry Letters* **2018,** *28* (3), 315-318.

20. Sun, S. S.; Ma, K.; Tao, Q. Q.; Han, J. J.; Bao, L.; Liu, L.; Liu, H. W., Diketopiperazines and 2H-pyran-2-ones with antioxidant activity from the rice fermented with Aspergillus luchuensis. *Fitoterapia* **2018,** *125*, 266-272.

21. Shin, H. J.; Choi, B. K.; Trinh, P. T. H.; Lee, H. S.; Kang, J. S.; Van, T. T. T.; Lee, H. S.; Lee, J. S.; Lee, Y. J.; Lee, J., Suppression of RANKL-Induced Osteoclastogenesis by the Metabolites from the Marine Fungus Aspergillus flocculosus Isolated from a Sponge Stylissa sp. *Marine Drugs* **2018,** *16* (1).

22. Qi, C. X.; Gao, W. X.; Guan, D. Y. Z.; Wang, J. P.; Liu, M. T.; Chen, C. M.; Zhu, H. C.; Zhou, Y.; Lai, Y. J.; Hu, Z. X.; Zhou, Q.; Zhang, Y. H., Butenolides from a marine-derived fungus Aspergillus terreus with antitumor activities against pancreatic ductal adenocarcinoma cells. *Bioorganic & Medicinal Chemistry* **2018,** *26* (22), 5903-5910.

23. Peng, X. P.; Wang, Y.; Zhu, T. H.; Zhu, W. M., Pyrazinone derivatives from the coral-derived Aspergillus ochraceus LCJ11-102 under high iodide salt. *Archives of Pharmacal Research* **2018,** *41* (2), 184-191.

24. Liu, M. T.; Sun, W. G.; Wang, J. P.; He, Y.; Zhang, J. W.; Li, F. L.; Qi, C. X.; Zhu, H. C.; Xue, Y. B.; Hu, Z. X.; Zhang, Y. H., Bioactive secondary metabolites from the marine-associated fungus Aspergillus terreus. *Bioorganic Chemistry* **2018,** *80*, 525-530.

25. Liu, J. T.; Gu, B. B.; Yang, L. J.; Yang, F.; Lin, H. W., New Anti-inflammatory Cyclopeptides From a Sponge-Derived Fungus Aspergillus violaceofuscus. *Frontiers in Chemistry* **2018,** *6*.

26. Limbadri, S.; Luo, X. W.; Lin, X. P.; Liao, S. R.; Wang, J. F.; Zhou, X. F.; Yang, B.; Liu, Y. H., Bioactive Novel Indole Alkaloids and Steroids from Deep Sea-Derived Fungus Aspergillus fumigatus SCSIO 41012. *Molecules* **2018,** *23* (9).

27. Li, Z. X.; Wang, X. F.; Ren, G. W.; Yuan, X. L.; Deng, N.; Ji, G. X.; Li, W.; Zhang, P., Prenylated Diphenyl Ethers from the Marine Algal-Derived Endophytic Fungus Aspergillus tennesseensis. *Molecules* **2018,** *23* (9).

28. Li, X. Y.; Ding, W. J.; Wang, P. M. M.; Xu, J. Z., Two Novel Aspochalasins from the Gut Fungus Aspergillus sp Z4. *Marine Drugs* **2018,** *16* (10).

29. Li, W. T.; Luo, D.; Huang, J. N.; Wang, L. L.; Zhang, F. G.; Xi, T.; Liao, J. M.; Lu, Y. Y., Antibacterial constituents from Antarctic fungus, Aspergillus sydowii SP-1. *Natural Product Research* **2018,** *32* (6), 662-667.

30. Li, H. Q.; Sun, W. G.; Deng, M. Y.; Zhou, Q.; Wang, J. P.; Liu, J. J.; Chen, C. M.; Qi, C. X.; Luo, Z. W.; Xue, Y. B.; Zhu, H. C.; Zhang, Y. H., Asperversiamides, Linearly Fused Prenylated Indole Alkaloids from the Marine-Derived Fungus Aspergillus versicolor. *Journal of Organic Chemistry* **2018,** *83* (15), 8483-8492.

31. Li, H. Q.; Sun, W. G.; Deng, M. Y.; Qi, C. X.; Chen, C. M.; Zhu, H. C.; Luo, Z. W.; Wang, J. P.; Xue, Y. B.; Zhang, Y. H., Asperversins A and B, Two Novel Meroterpenoids with an Unusual 5/6/6/6 Ring from the Marine-Derived Fungus Aspergillus versicolor. *Marine Drugs* **2018,** *16* (6).

32. Kong, F. D.; Huang, X. L.; Ma, Q. Y.; Xie, Q. Y.; Wang, P.; Chen, P. W.; Zhou, L. M.; Yuan, J. Z.; Dai, H. F.; Luo, D. Q.; Zhao, Y. X., Helvolic Acid Derivatives with Antibacterial Activities against Streptococcus agalactiae from the Marine-Derived Fungus Aspergillus fumigatus HNMF0047. *Journal of Natural Products* **2018,** *81* (8), 1869-1876.

33. Ivanets, E. V.; Yurchenko, A. N.; Smetanina, O. F.; Rasin, A. B.; Zhuravleva, O. I.; Pivkin, M. V.; Popov, R. S.; von Amsberg, G.; Afiyatullov, S. S.; Dyshlovoy, S. A., Asperindoles A-D and a p-Terphenyl Derivative from the Ascidian-Derived Fungus Aspergillus sp KMM 4676. *Marine Drugs* **2018,** *16* (7).

34. Guo, Z. K.; Zhou, Y. Q.; Han, H.; Wang, W.; Xiang, L.; Deng, X. Z.; Ge, H. M.; Jiao, R. H., New Antibacterial Phenone Derivatives Asperphenone A-C from Mangrove-Derived Fungus Aspergillus sp YHZ-1. *Marine Drugs* **2018,** *16* (2).

35. Gu, B. B.; Jiao, F. R.; Wu, W.; Jiao, W. H.; Li, L.; Sun, F.; Wang, S. P.; Yang, F.; Lin, H. W., Preussins with Inhibition of IL-6 Expression from Aspergillus flocculosus 16D-1, a Fungus Isolated from the Marine Sponge Phakellia fusca. *Journal of Natural Products* **2018,** *81* (10), 2275-2281.

36. Ebada, S. S.; El-Neketi, M.; Ebrahim, W.; Mandi, A.; Kurtan, T.; Kalscheuer, R.; Muller, W. E. G.; Proksch, P., Cytotoxic secondary metabolites from the endophytic fungus Aspergillus versicolor KU258497. *Phytochemistry Letters* **2018,** *24,* 88-93.

37. Du, X. W.; Liu, D.; Huang, J.; Zhang, C. J.; Proksch, P.; Lin, W. H., Polyketide derivatives from the sponge associated fungus Aspergillus europaeus with antioxidant and NO inhibitory activities. *Fitoterapia* **2018,** *130*, 190-197.

38. Cho, K. H.; Sohn, J. H.; Oh, H., Isolation and structure determination of a new diketopiperazine dimer from marine-derived fungus Aspergillus sp SF-5280. *Natural Product Research* **2018,** *32* (2), 214-221.

39. Cheng, Z. B.; Liu, D.; Cheng, W.; Proksch, P.; Lin, W. H., Versiquinazolines L-Q, new polycyclic alkaloids from the marine-derived fungus Aspergillus versicolor. *Rsc Advances* **2018,** *8* (55), 31427-31439.

40. Buttachon, S.; Ramos, A. A.; Inacio, A.; Dethoup, T.; Gales, L.; Lee, M.; Costa, P. M.; Silva, A. M. S.; Sekeroglu, N.; Rocha, E.; Pinto, M. M. M.; Pereira, J. A.; Kijjoa, A., Bis-Indolyl Benzenoids, Hydroxypyrrolidine Derivatives and Other Constituents from Cultures of the Marine

Sponge-Associated Fungus Aspergillus candidus KUFA0062. *Marine Drugs* **2018,** *16* (4).

41. Afiyatullov, S. S.; Zhuravleva, O. I.; Antonov, A. S.; Berdyshev, D. V.; Pivkin, M. V.; Denisenko, V. A.; Popov, R. S.; Gerasimenko, A. V.; von Amsberg, G.; Dyshlovoy, S. A.; Leshchenko, E. V.; Yurchenko, A. N., Prenylated indole alkaloids from co-culture of marine-derived fungi Aspergillus sulphureus and Isaria felina. *Journal of Antibiotics* **2018,** *71* (10), 846-853.

42. Yan, W.; Wuringege; Li, S. J.; Guo, Z. K.; Zhang, W. J.; Wei, W.; Tan, R. X.; Jiao, R. H., New p-terphenyls from the endophytic fungus Aspergillus sp YXf3. *Bioorganic & Medicinal Chemistry Letters* **2017,** *27* (1), 51-54.

43. Yamada, T.; Kimura, H.; Arimitsu, K.; Kajimoto, T.; Kikuchi, T.; Tanaka, R., Absolute Configuration of Eight Cephalimysins Isolated from the Marine-Derived Aspergillus fumigatus. *Chemistryselect* **2017,** *2* (33), 10936-10940.

44. Xu, X. L.; Zhao, S. J.; Yin, L. Y.; Yu, Y.; Chen, Z. K.; Shen, H. H.; Zhou, L., A New Sydonic Acid Derivative From a Marine Derived-Fungus Aspergillus sydowii. *Chemistry of Natural Compounds* **2017,** *53* (6), 1056-1058.

45. Xu, L. L.; Zhang, C. C.; Zhu, X. Y.; Cao, F.; Zhu, H. J., Bioactive phenyl ether derivatives from the marine-derived fungus Aspergillus carneus. *Natural Product Research* **2017,** *31* (16), 1875-1879.

46. Wang, Y.; Lin, X. P.; Ju, Z. R.; Liao, X. J.; Huang, X. J.; Zhang, C.; Zhao, B. X.; Xu, S. H., Aspergchromones A and B, two new polyketides from the marine sponge-associated fungus Aspergillus sp SCSIO XWS03F03. *Journal of Asian Natural Products Research* **2017,** *19* (7), 684-690.

47. Uchoa, P. K. S.; Pimenta, A. T. A.; Braz, R.; de Oliveira, M. D. F.; Saraiva, N. N.; Rodrigues, B. S. F.; Pfenning, L. H.; Abreu, L. M.; Wilke, D. V.; Florenciog, K. G. D.; Lima, M. A. S., New cytotoxic furan from the marine sediment-derived fungi Aspergillus niger. *Natural Product Research* **2017,** *31* (22), 2599-2603.

48. Sugimoto, K.; Sadahiro, Y.; Kagiyama, I.; Kato, H.; Sherman, D. H.; Williams, R. M.; Tsukamoto, S., Isolation of amoenamide A and five antipodal prenylated alkaloids from Aspergillus amoenus NRRL 35600. *Tetrahedron Letters* **2017,** *58* (29), 2797-2800.

49. Ma, X.; Nong, X. H.; Ren, Z.; Wang, J.; Liang, X.; Wang, L.; Qi, S. H., Antiviral peptides from marine gorgonian-derived fungus Aspergillus sp SCSIO 41501. *Tetrahedron Letters* **2017,** *58* (12), 1151-1155.

50. Liu, S.; Wang, H.; Su, M.; Hwang, G. J.; Hong, J.; Jung, J. H., New metabolites from the sponge-derived fungus Aspergillus sydowii J05B-7F-4. *Natural Product Research* **2017,** *31* (14), 1682-1686.

51. Liao, L. J.; Bae, S. Y.; Won, T. H.; You, M. J.; Kim, S. H.; Oh, D. C.; Lee, S. K.; Oh, K. B.; Shin, J., Asperphenins A and B, Lipopeptidyl Benzophenones from a Marine-Derived Aspergillus sp Fungus. *Organic Letters* **2017,** *19* (8), 2066-2069.

52. Li, X. Y.; Zhao, Z. H.; Ding, W. J.; Ye, B.; Wang, P. M.; Xu, J. Z., Aspochalazine A, a novel polycyclic aspochalasin from the fungus Aspergillus sp Z4. *Tetrahedron Letters* **2017,** *58* (25), 2405-2408.

53. Li, X. F.; Xia, Z. Y.; Tang, J. Q.; Wu, J. H.; Tong, J.; Li, M. J.; Ju, J. H.; Chen, H. R.; Wang, L. Y., Identification and Biological Evaluation of Secondary Metabolites from Marine Derived Fungi-Aspergillus sp SCSIOW3, Cultivated in the Presence of Epigenetic Modifying Agents. *Molecules* **2017,** *22* (8).

54. Kwon, J.; Lee, H.; Ko, W.; Kim, D. C.; Kim, K. W.; Kwon, H. C.; Guo, Y. Q.; Sohn, J. H.; Yim, J. H.; Kim, Y. C.; Oh, H.; Lee, D., Chemical constituents isolated from Antarctic marine-derived Aspergillus sp SF-5976 and their anti-inflammatory effects in LPS-stimulated RAW 264.7 and BV2 cells. *Tetrahedron* **2017,** *73* (27-28), 3905-3912.

55. Hou, X. M.; Zhang, Y. H.; Hai, Y.; Zheng, J. Y.; Gu, Y. C.; Wang, C. Y.; Shao, C. L., Aspersymmetide A, a New Centrosymmetric Cyclohexapeptide from the Marine-Derived Fungus Aspergillus versicolor. *Marine Drugs* **2017,** *15* (11).

56. Guo, Z. K.; Gai, C. J.; Cai, C. H.; Chen, L. L.; Liu, S. B.; Zeng, Y. B.; Yuan, J. Z.; Mei, W. L.; Dai, H. F., Metabolites with Insecticidal Activity from Aspergillus fumigatus JRJ111048 Isolated from Mangrove Plant Acrostichum specioum Endemic to Hainan Island. *Marine Drugs* **2017,** *15* (12).

57. Bao, J.; Wang, J.; Zhang, X. Y.; Nong, X. H.; Qi, S. H., New Furanone Derivatives and Alkaloids from the Co-Culture of Marine-Derived Fungi Aspergillus sclerotiorum and Penicillium citrinum. *Chemistry & Biodiversity* **2017,** *14* (3).

58. Aparicio-Cuevas, M. A.; Rivero-Cruz, I.; Sanchez-Castellanos, M.; Menendez, D.; Raja, H. A.; Joseph-Nathan, P.; Gonzalez, M. D.; Figueroa, M., Dioxomorpholines and Derivatives from a Marine-Facultative Aspergillus Species. *Journal of Natural Products* **2017,** *80* (8), 2311-2318.

59. Zhuravleva, O. I.; Kirichuk, N. N.; Denisenko, V. A.; Dmitrenok, P. S.; Pivkin, M. V.; Afiyatullov, S. S., New Kipukasin from Marine Isolate of the Fungus Aspergillus flavus. *Chemistry of Natural Compounds* **2016,** *52* (2), 266-268.

60. Zhou, X. F.; Fang, W.; Tan, S. Y.; Lin, X. P.; Xun, T. R.; Yang, B. J.; Liu, S. W.; Liu, Y. H., Aspernigrins with anti-HIV-1 activities from the marine-derived fungus Aspergillus niger SCSIO Jcsw6F30. *Bioorganic & Medicinal Chemistry Letters* **2016,** *26* (2), 361-365.

61. Zhou, X.; Fang, P. Y.; Tang, J. Q.; Wu, Z. Q.; Li, X. F.; Li, S. M.; Wang, Y.; Liu, G.; He, Z. D.; Gou, D. M.; Yao, X. S.; Wang, L. Y., A novel cyclic dipeptide from deep marine-derived fungus Aspergillus sp SCSIOW2. *Natural Product Research* **2016,** *30* (1), 52-57.

62. Wu, Z. H.; Wang, Y. R.; Liu, D.; Proksch, P.; Yu, S. W.; Lin, W. H., Antioxidative phenolic compounds from a marine-derived fungus Aspergillus versicolor. *Tetrahedron* **2016,** *72* (1), 50-57.

63. Wang, Y.; Li, D. H.; Li, Z. L.; Sun, Y. J.; Hua, H. M.; Liu, T.; Bai, J., Terpenoids from the Marine-Derived Fungus Aspergillus fumigatus YK-7. *Molecules* **2016,** *21* (1).

64. Wang, L. Y.; Li, M. J.; Tang, J. Q.; Li, X. F., Eremophilane Sesquiterpenes from a Deep Marine-Derived Fungus, Aspergillus sp SCSIOW2, Cultivated in the Presence of Epigenetic Modifying Agents. *Molecules* **2016,** *21* (4).

65. Wang, C.; Guo, L.; Hao, J. J.; Wang, L. P.; Zhu, W. M., alpha-Glucosidase Inhibitors from the Marine-Derived Fungus Aspergillus flavipes HN4-13. *Journal of Natural Products* **2016,** *79* (11), 2977-2981.

66. Tian, Y. Q.; Lin, X. P.; Wang, Z.; Zhou, X. F.; Qin, X. C.; Kaliyaperumal, K.; Zhang, T. Y.; Tu, Z. C.; Liu, Y. H., Asteltoxins with Antiviral Activities from the Marine Sponge-Derived Fungus Aspergillus sp SCSIO XWS02F40. *Molecules* **2016,** *21* (1).

67. Oleinikova, G. K.; Denisenko, V. A.; Berdyshev, D. V.; Pushilin, M. A.; Kirichuk, N. N.; Menzorova, N. I.; Kuzmich, A. S.; Yurchenko, E. A.; Zhuravleva, O. I.; Afiyatullov, S. S., Two new sesterterpenoids, terretonins H and I, from the marine-derived fungus Aspergillus ustus. *Phytochemistry*

Letters **2016,** *17*, 135-139.

68. Liu, Z. M.; Chen, Y.; Chen, S. H.; Liu, Y. Y.; Lu, Y. J.; Chen, D. N.; Lin, Y. C.; Huang, X. S.; She, Z. G., Aspterpenacids A and B, Two Sesterterpenoids from a Mangrove Endophytic Fungus Aspergillus terreus H010. *Organic Letters* **2016,** *18* (6), 1406-1409.

69. Liu, S.; Dai, H.; Konuklugil, B.; Orfali, R. S.; Lin, W. H.; Kalscheuer, R.; Liu, Z.; Proksch, P., Phenolic bisabolanes from the sponge-derived fungus Aspergillus sp. *Phytochemistry Letters* **2016,** *18*, 187-191.

70. Li, X. D.; Li, X. M.; Li, X.; Xu, G. M.; Liu, Y.; Wang, B. G., Aspewentins D-H, 20-Nor-isopimarane Derivatives from the Deep Sea Sediment-Derived Fungus Aspergillus wentii SD-310. *Journal of Natural Products* **2016,** *79* (5), 1347-1353.

71. Li, X. D.; Li, X.; Li, X. M.; Xu, G. M.; Zhang, P.; Meng, L. H.; Wang, B. G., Tetranorlabdane Diterpenoids from the Deep Sea Sediment-Derived Fungus Aspergillus wentii SD-310. *Planta Medica* **2016,** *82* (9-10), 877-881.

72. Li, T. X.; Yang, M. H.; Wang, Y.; Wang, X. B.; Luo, J.; Luo, J. G.; Kong, L. Y., Unusual dimeric tetrahydroxanthone derivatives from Aspergillus lentulus and the determination of their axial chiralities. *Scientific Reports* **2016,** *6*.

73. Li, D. H.; Han, T.; Guan, L. P.; Bai, J.; Zhao, N.; Li, Z. L.; Wu, X.; Hua, H. M., New naphthopyrones from marine-derived fungus Aspergillus niger 2HL-M-8 and their in vitro antiproliferative activity. *Natural Product Research* **2016,** *30* (10), 1116-1122.

74. Kagiyama, I.; Kato, H.; Nehira, T.; Frisvad, J. C.; Sherman, D. H.; Williams, R. M.; Tsukamoto, S., Taichunamides: Prenylated Indole Alkaloids from Aspergillus taichungensis (IBT 19404). *Angewandte Chemie-International Edition* **2016,** *55* (3), 1128-1132.

75. Ding, C. H.; Wu, X. D.; Auckloo, B. N.; Chen, C. T. A.; Ye, Y.; Wang, K. W.; Wu, B., An Unusual Stress Metabolite from a Hydrothermal Vent Fungus Aspergillus sp WU 243 Induced by Cobalt. *Molecules* **2016,** *21* (1).

76. Zheng, C. J.; Wu, L. Y.; Li, X. B.; Song, X. M.; Niu, Z. G.; Song, X. P.; Chen, G. Y.; Wang, C. Y., Structure and Absolute Configuration of Aspergilumamide A, a Novel Lumazine Peptide from the Mangrove-Derived Fungus Aspergillus sp. *Helvetica Chimica Acta* **2015,** *98* (3), 368-373.

77. Zhang, P.; Li, X. M.; Li, X.; Wang, B. G., New indole-diterpenoids from the algal-associated fungus Aspergillus nidulans. *Phytochemistry Letters* **2015,** *12*, 182-185.

78. Yurchenko, A. A.; Smetanina, O. F.; Kalinovsky, A. I.; Kirichuk, N. N.; Pivkin, M. V.; Ivanets, E. V.; Yurchenko, E. A.; Afiyatullov, S. S., New Metabolites from a Marine Sediment-Derived Fungus, Aspergillus carneus. *Natural Product Communications* **2015,** *10* (7), 1247-1250.

79. You, M.; Liao, L.; Hong, S. H.; Park, W.; Kwon, D. I.; Lee, J.; Noh, M.; Oh, D. C.; Oh, K. B.; Shin, J., Lumazine Peptides from the Marine-Derived Fungus Aspergillus terreus. *Marine Drugs* **2015,** *13* (3), 1290-1303.

80. Xiao, Z. E.; Lin, S. E.; Tan, C. B.; Lu, Y. J.; He, L.; Huang, X. S.; She, Z. G., Asperlones A and B, Dinaphthalenone Derivatives from a Mangrove Endophytic Fungus Aspergillus sp 16-5C. *Marine Drugs* **2015,** *13* (1), 366-378.

81. Wu, G. W.; Yu, G. H.; Kurtan, T.; Mandi, A.; Peng, J. X.; Mo, X. M.; Liu, M.; Li, H.; Sun, X. H.; Li, J.; Zhu, T. J.; Gu, Q. Q.; Li, D. H., Versixanthones A-F, Cytotoxic

Xanthone-Chromanone Dimers from the Marine-Derived Fungus Aspergillus versicolor HDN1009. *Journal of Natural Products* **2015,** *78* (11), 2691-2698.

82. Wang, Y. Z.; Qi, S.; Zhan, Y.; Zhang, N. W.; Wu, A. A.; Gui, F.; Guo, K.; Yang, Y. R.; Cao, S. G.; Hu, Z. Y.; Zheng, Z. H.; Song, S. Y.; Xu, Q. Y.; Shen, Y. M.; Deng, X. M., Aspertetranones A-D, Putative Meroterpenoids from the Marine Algal-Associated Fungus Aspergillus sp ZL0-1b14. *Journal of Natural Products* **2015,** *78* (10), 2405-2410.

83. Wang, P. M.; Zhao, S. Z.; Liu, Y.; Ding, W. J.; Qiu, F.; Xu, J. Z., Asperginine, an Unprecedented Alkaloid from the Marine-derived Fungus Aspergillus sp. *Natural Product Communications* **2015,** *10* (8), 1363-1364.

84. Shi, Y. S.; Zhang, Y.; Chen, X. Z.; Zhang, N.; Liu, Y. B., Metabolites Produced by the Endophytic Fungus Aspergillus fumigatus from the Stem of Erythrophloeum fordii Oliv. *Molecules* **2015,** *20* (6), 10793-10799.

85. Prompanya, C.; Fernandes, C.; Cravo, S.; Pinto, M. M. M.; Dethoup, T.; Silva, A. M. S.; Kijjoa, A., A New Cyclic Hexapeptide and a New Isocoumarin Derivative from the Marine Sponge-Associated Fungus Aspergillus similanensis KUFA 0013. *Marine Drugs* **2015,** *13* (3), 1432-1450.

86. Nong, X. H.; Zhang, X. Y.; Xu, X. Y.; Qi, S. H., Antifouling Compounds from the Marine-Derived Fungus Aspergillus terreus SCSGAF0162. *Natural Product Communications* **2015,** *10* (6), 1033-1034.

87. Liu, Y. Y.; Chen, S. H.; Liu, Z. M.; Lu, Y. J.; Xia, G. P.; Liu, H. J.; He, L.; She, Z. G., Bioactive Metabolites from Mangrove Endophytic Fungus Aspergillus sp 16-5B. *Marine Drugs* **2015,** *13* (5), 3091-3102.

88. Liu, Y.; Li, X. M.; Meng, L. H.; Wang, B. G., Polyketides from the marine mangrove-derived fungus Aspergillus ochraceus MA-15 and their activity against aquatic pathogenic bacteria. *Phytochemistry Letters* **2015,** *12*, 232-236.

89. Liao, L.; You, M.; Chung, B. K.; Oh, D. C.; Oh, K. B.; Shin, J., Alkaloidal Metabolites from a Marine-Derived Aspergillus sp Fungus. *Journal of Natural Products* **2015,** *78* (3), 349-354.

90. Li, X. B.; Zhou, Y. H.; Zhu, R. X.; Chang, W. Q.; Yuan, H. Q.; Gao, W.; Zhang, L. L.; Zhao, Z. T.; Lou, H. X., Identification and Biological Evaluation of Secondary Metabolites from the Endolichenic Fungus Aspergillus versicolor. *Chemistry & Biodiversity* **2015,** *12* (4), 575-592.

91. Kong, F. D.; Zhao, C. Y.; Hao, J. J.; Wang, C.; Wang, W.; Huang, X. L.; Zhu, W. M., New alpha-glucosidase inhibitors from a marine sponge-derived fungus, Aspergillus sp OUCMDZ-1583. *Rsc Advances* **2015,** *5* (84), 68852-68863.

92. Happi, G. M.; Kouam, S. F.; Talontsi, F. M.; Nkenfou, C. N.; Longo, F.; Zuhlke, S.; Douanla-Meli, C.; Spiteller, M., A new dimeric naphtho-gamma-pyrone from an endophytic fungus Aspergillus niger AKRN associated with the roots of Entandrophragma congoense collected in Cameroon. *Zeitschrift Fur Naturforschung Section B-a Journal of Chemical Sciences* **2015,** *70* (9), 625-630.

93. Gao, Y. Q.; Guo, C. J.; Zhang, Q.; Zhou, W. M.; Wang, C. C. C.; Gao, J. M., Asperaculanes A and B, Two Sesquiterpenoids from the Fungus Aspergillus aculeatus. *Molecules* **2015,** *20* (1), 325-334.

94. Fredimoses, M.; Zhou, X. F.; Ai, W.; Tian, X. P.; Yang, B.; Lin, X. P.; Xian, J. Y.; Liu, Y. H., Westerdijkin A, a new hydroxyphenylacetic acid derivative from deep sea fungus Aspergillus westerdijkiae SCSIO 05233. *Natural Product Research* **2015,** *29* (2), 158-162.

95. Chen, M.; Wang, K. L.; Liu, M.; She, Z. G.; Wang, C. Y., Bioactive Steroid Derivatives and

Butyrolactone Derivatives from a Gorgonian-Derived Aspergillus sp Fungus. *Chemistry & Biodiversity* **2015,** *12* (9), 1398-1406.

96. Cardoso-Martinez, F.; de la Rosa, J. M.; Diaz-Marrero, A. R.; Darias, J.; D'Croz, L.; Cerella, C.; Diederich, M.; Cueto, M., Oximoaspergillimide, a Fungal Derivative from a Marine Isolate of Aspergillus sp. *European Journal of Organic Chemistry* **2015,** (10), 2256-2261.

97. Zhu, T. H.; Chen, Z. Q.; Liu, P. P.; Wang, Y.; Xin, Z. H.; Zhu, W. M., New rubrolides from the marine-derived fungus Aspergillus terreus OUCMDZ-1925. *Journal of Antibiotics* **2014,** *67* (4), 315-318.

98. Zhou, Y. M.; Debbab, A.; Wray, V.; Lin, W. H.; Schulz, B.; Trepos, R.; Pile, C.; Hellio, C.; Proksch, P.; Aly, A. H., Marine bacterial inhibitors from the sponge-derived fungus Aspergillus sp. *Tetrahedron Letters* **2014,** *55* (17), 2789-2792.

99. Wang, J. F.; Lin, X. P.; Qin, C.; Liao, S. R.; Wan, J. T.; Zhang, T. Y.; Liu, J.; Fredimoses, M.; Chen, H.; Yang, B.; Zhou, X. F.; Yang, X. W.; Tu, Z. C.; Liu, Y. H., Antimicrobial and antiviral sesquiterpenoids from sponge-associated fungus, Aspergillus sydowii ZSDS1-F6. *Journal of Antibiotics* **2014,** *67* (8), 581-583.

100. Tang, Q.; Guo, K.; Li, X. Y.; Zheng, X. Y.; Kong, X. J.; Zheng, Z. H.; Xu, Q. Y.; Deng, X. M., Three New Asperentin Derivatives from the Algicolous Fungus Aspergillus sp F00785. *Marine Drugs* **2014,** *12* (12), 5993-6002.

101. Sun, K. L.; Li, Y.; Guo, L.; Wang, Y.; Liu, P. P.; Zhu, W. M., Indole Diterpenoids and Isocoumarin from the Fungus, Aspergillus flavus, Isolated from the Prawn, Penaeus vannamei. *Marine Drugs* **2014,** *12* (7), 3970-3981.

102. Song, F. H.; Ren, B.; Chen, C. X.; Yu, K.; Liu, X. R.; Zhang, Y. H.; Yang, N.; He, H. T.; Liu, X. T.; Dai, H. Q.; Zhang, L. X., Three new sterigmatocystin analogues from marine-derived fungus Aspergillus versicolor MF359. *Applied Microbiology and Biotechnology* **2014,** *98* (8), 3753-3758.

103. Rukachaisirikul, V.; Rungsaiwattana, N.; Klaiklay, S.; Phongpaichit, S.; Borwomwiriyapan, K.; Sakayaroji, J., gamma-Butyrolactone, Cytochalasin, Cyclic Carbonate, Eutypinic Acid, and Phenalenone Derivatives from the Soil Fungus Aspergillus sp PSU-RSPG185. *Journal of Natural Products* **2014,** *77* (11), 2375-2382.

104.Prompanya, C.; Dethoup, T.; Bessa, L. J.; Pinto, M. M. M.; Gales, L.; Costa, P. M.; Silva, A. M. S.; Kijjoa, A., New Isocoumarin Derivatives and Meroterpenoids from the Marine Sponge-Associated Fungus Aspergillus similanensis sp nov KUFA 0013. *Marine Drugs* **2014,** *12* (10), 5160-5173.

105. Petersen, L. M.; Hoeck, C.; Frisvad, J. C.; Gotfredsen, C. H.; Larsen, T. O., Dereplication Guided Discovery of Secondary Metabolites of Mixed Biosynthetic Origin from Aspergillus aculeatus. *Molecules* **2014,** *19* (8), 10898-10921.

106. Peng, J. X.; Gao, H. Q.; Zhang, X. M.; Wang, S.; Wu, C. M.; Gu, Q. Q.; Guo, P.; Zhu, T. J.; Li, D. H., Psychrophilins E-H and Versicotide C, Cyclic Peptides from the Marine-Derived Fungus Aspergillus versicolor ZLN-60. *Journal of Natural Products* **2014,** *77* (10), 2218-2223.

107. Peng, J. X.; Gao, H. Q.; Li, J.; Ai, J.; Geng, M. Y.; Zhang, G. J.; Zhu, T. J.; Gu, Q. Q.; Li, D. H., Prenylated Indole Diketopiperazines from the Marine-Derived Fungus Aspergillus versicolor. *Journal of Organic Chemistry* **2014,** *79* (17), 7895-7904.

108. Nong, X. H.; Wang, Y. F.; Zhang, X. Y.; Zhou, M. P.; Xu, X. Y.; Qi, S. H., Territrem and Butyrolactone Derivatives from a Marine-Derived Fungus Aspergillus Terreus. *Marine Drugs* **2014,** *12* (12),

6113-6124.

109. Miao, F. P.; Liang, X. R.; Liu, X. H.; Ji, N. Y., Aspewentins A-C, Norditerpenes from a Cryptic Pathway in an Algicolous Strain of Aspergillus wentii. *Journal of Natural Products* **2014,** *77* (2), 429-432.

110. Ma, X. H.; Zhu, T. J.; Gu, Q. Q.; Xi, R.; Wang, W.; Li, D. H., Structures and antiviral activities of butyrolactone derivatives isolated from Aspergillus terreus MXH-23. *Journal of Ocean University of China* **2014,** *13* (6), 1067-1070.

111. Liu, Y.; Zhao, S. Z.; Ding, W. J.; Wang, P. M.; Yang, X. W.; Xu, J. Z., Methylthio-Aspochalasins from a Marine-Derived Fungus Aspergillus sp. *Marine Drugs* **2014,** *12* (10), 5124-5131.

112. Liu, X. H.; Miao, F. P.; Liang, X. R.; Ji, N. Y., Ergosteroid derivatives from an algicolous strain of Aspergillus ustus. *Natural Product Research* **2014,** *28* (15), 1182-1186.

113. Li, X.; Li, X. M.; Xu, G. M.; Li, C. S.; Wang, B. G., Antioxidant metabolites from marine alga-derived fungus Aspergillus wentii EN-48. *Phytochemistry Letters* **2014,** *7*, 120-123.

114. Khalil, Z. G.; Huang, X. C.; Raju, R.; Piggott, A. M.; Capon, R. J., Shornephine A: Structure, Chemical Stability, and P-Glycoprotein Inhibitory Properties of a Rare Diketomorpholine from an Australian Marine-Derived Aspergillus sp. *Journal of Organic Chemistry* **2014,** *79* (18), 8700-8705.

115. Hu, X.; Xia, Q. W.; Zhao, Y. Y.; Zheng, Q. H.; Liu, Q. Y.; Chen, L.; Zhang, Q. Q., SPERADINES B-E, FOUR NOVEL TETRACYCLIC OXINDOLE ALKALOIDS FROM THE MARINE-DERIVED FUNGUS ASPERGILLUS ORYZAE. *Heterocycles* **2014,** *89* (7), 1662-1669.

116. Hu, X.; Xia, Q. W.; Zhao, Y. Y.; Zheng, Q. H.; Liu, Q. Y.; Chen, L.; Zhang, Q. Q., Speradines F-H, Three New Oxindole Alkaloids from the Marine-Derived Fungus Aspergillus oryzae. *Chemical & Pharmaceutical Bulletin* **2014,** *62* (9), 942-946.

117. Fukuda, T.; Kurihara, Y.; Kanamoto, A.; Tomoda, H., Terretonin G, a new sesterterpenoid antibiotic from marine-derived Aspergillus sp OPMF00272. *Journal of Antibiotics* **2014,** *67* (8), 593-595.

118. Fang, W.; Lin, X. P.; Zhou, X. F.; Wan, J. T.; Lu, X.; Yang, B.; Ai, W.; Lin, J.; Zhang, T. Y.; Tu, Z. C.; Liu, Y. H., Cytotoxic and antiviral nitrobenzoyl sesquiterpenoids from the marine-derived fungus Aspergillus ochraceus Jcma1F17. *Medchemcomm* **2014,** *5* (6), 701-705.

119. Ebada, S. S.; Fischer, T.; Hamacher, A.; Du, F. Y.; Roth, Y. O.; Kassack, M. U.; Wang, B. G.; Roth, E. H., Psychrophilin E, a new cyclotripeptide, from co-fermentation of two marine alga-derived fungi of the genus Aspergillus. *Natural Product Research* **2014,** *28* (11), 776-781.

120. Chen, X. W.; Li, C. W.; Cui, C. B.; Hua, W.; Zhu, T. J.; Gu, Q. Q., Nine New and Five Known Polyketides Derived from a Deep Sea-Sourced Aspergillus sp 16-02-1. *Marine Drugs* **2014,** *12* (6), 3116-3137.

121. Chen, M.; Shao, C. L.; Meng, H.; She, Z. G.; Wang, C. Y., Anti-Respiratory Syncytial Virus Prenylated Dihydroquinolone Derivatives from the Gorgonian-Derived Fungus Aspergillus sp. XS-20090B15. *Journal of Natural Products* **2014,** *77* (12), 2720-2724.

122. Chen, M.; Fu, X. M.; Kong, C. J.; Wang, C. Y., Nucleoside derivatives from the marine-derived fungus Aspergillus versicolor. *Natural Product Research* **2014,** *28* (12), 895-900.

123. Bai, Z. Q.; Lin, X. P.; Wang, Y. Z.; Wang, J. F.; Zhou, X. F.; Yang, B.; Liu, J.; Yang, X. W.; Wang, Y.; Liu, Y. H., New phenyl derivatives from endophytic fungus Aspergillus flavipes AIL8 derived of mangrove plant Acanthus ilicifolius. *Fitoterapia* **2014,** *95*, 194-202.
